# Inheritance of Black Rot Resistance and Development of Molecular Marker Linked to *Xcc* Races 6 and 7 Resistance in Cabbage

**DOI:** 10.3390/plants10091940

**Published:** 2021-09-17

**Authors:** Jeong-Eui Hong, Khandker Shazia Afrin, Md Abdur Rahim, Hee-Jeong Jung, Ill-Sup Nou

**Affiliations:** 1 Department of Horticulture, Sunchon National University, 255 Jungang-ro, Suncheon 57922, Korea; gns1017@naver.com (J.-E.H.); raidahnoshaba@gmail.com (K.S.A.); 2 Department of Genetics and Plant Breeding, Sher-e-Bangla Agricultural University, Dhaka 1207, Bangladesh; rahimgepb@sau.edu.bd

**Keywords:** *Xcc*, race 6/7, black rot, molecular marker, cabbage

## Abstract

Black rot, caused by *Xanthomonas campestris* pv. *campestris* (*Xcc*), produces V-shaped chlorotic lesions on the leaves of cabbage (*Brassica oleracea* var. *capitata* L.), causing darkened veins and drastically reducing yield and quality. Of the 11 *Xcc* races identified, races 1, 4, and 6 are predominant globally. In the present study, we aimed to develop a molecular marker linked to black rot resistance against *Xcc* races 6 and 7. Crossed between black rot-resistant (‘SCNU-C-3470’) and -susceptible (‘SCNU-C-3328’) lines obtained 186 F_2_ plants. Resistance to *Xcc* race 6 segregated in a 3:1 (susceptible:resistant) ratio in the F_2_ population, which is consistent with a monogenic recessive trait. Nucleotide-binding site (NBS) leucine rich repeat (LRR)-encoding resistance (*R*) genes play a crucial role in plant defenses to various pathogens. The candidate *R* gene (*Bol031422*) located on chromosome C08, previously reported by our research group, was cloned and sequenced in resistant and susceptible cabbage lines. The *R* gene *Bol031422* consisted of a single exon with a 3 bp insertion/deletions (InDels), a 292 bp polymorphism (an insertion in the exon of the resistant line relative to the susceptible line) and several single nucleotide polymorphisms (SNPs). Here, we developed the InDel marker BR6-InDel to assess linkage between variation at *Bol031422* and resistance to *Xcc* races 6 and 7. This marker will help cabbage breeders develop cabbage cultivars resistant to *Xcc* races 6 and 7.

## 1. Introduction

Cabbage (*Brassica oleracea* var. *capitata* L.) is an important vegetable in *Brassica* crops worldwide due to its high nutritional quality, good storage properties, and potential health benefits [1,2]. Black rot is the most devastating disease of *Brassica* vegetables, caused by the aerobic, Gram-negative, and nonsporulating bacterium *Xanthomonas campestris* pv. *campestris* (*Xcc*) [3]. Black rot is mostly a seed-borne and vascular disease [4,5] that can be spread via wind, rainfall, and insects [6]. Typical black rot symptoms include necrotic, darkened leaf veins and a V-shaped chlorotic lesion starting from the leaf margin [3,5]. Black rot substantially reduces the yield and quality of cabbage harvests. To date, 11 pathogenic *Xcc* races have been identified that can infect *Brassica* crops [7,8,9]. Among them, races 1, 4, and 6 are the most widespread worldwide and the most aggressive against *B. oleracea* crops [10].

The occurrence of black rot has also increased in frequency due to recent climate change trends, making it more difficult to mitigate the pathogen effectively via chemical control methods alone. Another, more ecologically benign means to control plant pathogens, especially fungal, bacterial, and viral diseases, is to identify and cultivate resistant cultivars, which can provide very effective control without the need for chemical treatments [11,12]. The cabbage cultivars ‘Early Fuji’ and ‘Hugenot’ were the first reported to be resistant to *Xcc* [13,14]. Breeders and researchers have also selected resistant genotypes and transferred the underlying resistance loci into other *Brassica* crops, including Ethiopian mustard (*Brassica carinata*), brown mustard (*Brassica juncea*), rapeseed (*Brassica napus*), black mustard (*Brassica nigra*), and field mustard (*Brassica rapa*), as well as cabbage. However, there are no known cabbage cultivars presenting resistance to *Xcc* in Korea, largely because cabbage cultivars that are resistant to black rot are not adapted to the local environment [9,15,16], creating a pressing need for studies on black rot resistance in cabbage. The development of resistant cabbage cultivars will require the identification and characterization of resistant cabbage germplasm as well as the gene(s) responsible for resistance to black rot.

We previously screened a collection of cabbage germplasm for resistance to black rot, resulting in the identification of resistant cabbage lines as well as *R* genes encoding nucleotide binding site (NBS) receptors linked to *Xcc* resistance [17,18]. In plants, most *R* genes encode NBS-site-leucine rich repeat (NBS-LRR) proteins [19,20]. Previous studies showed that the *R* gene *Bol037156* (Resistance gene to Race 1 of *Fusarium oxysporum* f. sp. *conglutinans*, *FOC1*) confers resistance to the fungal pathogen *Fusarium* in *B. oleracea* [21], whereas *RESISTANT TO P. SYRINGAE 6* (*RPS6*) controls resistance to *Pseudomonas syringae* in Arabidopsis (*Arabidopsis thaliana*) [22]. NBS-LRR-type *R* genes are critical for plant responses to various pathogens, including nematodes, bacteria, fungi, and viruses [23]. Recent studies have shown that *R* genes have also been identified in other plant species, such as *Arabidopsis thaliana*, melon (*Cucumis melo*), and rice (*Oryza sativa*) [24,25,26].

Molecular markers facilitate plant selection based on their genotype without the need for time-consuming phenotypic tests. These genotyping efforts typically rely on sequence-based molecular markers such as simple sequence repeats (SSRs) and single nucleotide polymorphisms (SNPs), as well as markers based on insertion/deletions (InDels). Our previous study showed susceptibility or resistance to *Xcc* of 27 cabbage inbred lines [18], highlighting lines ‘SCNU-C-3470’ and ‘SCNU-C-3328’ as being resistant and susceptible to *Xcc* races 6 and 7, respectively. Our recent study identified 32 differentially expressed *R* genes distributed across the *B. oleracea* genome [17]. Among these, one candidate gene (*Bol031422*) with higher expression in the resistant line than in susceptible line and higher expression in leaves than in other tissues was selected for further characterization. Accordingly, we sequenced the genomic region of *Bol031422* and identified several InDels between resistant and susceptible lines. Although efforts have been made to identify *R* genes conferring resistance to the pathogen [1,17] and develop methods to screen for resistance, very little is known about *Xcc*-race-specific *R* genes. In this study, we therefore aimed to develop a specific molecular marker to genotype plants for *Bol031422* and then compare these results to tests for resistance or susceptibility to *Xcc* races 6 and 7 by routine PCR to assess the contribution of *Bol031422* to the observed resistance to *Xcc*.

## 2. Results

### 2.1. Inheritance of Xcc Races 6 and 7 Resistance in Cabbage

**Figure 1:**
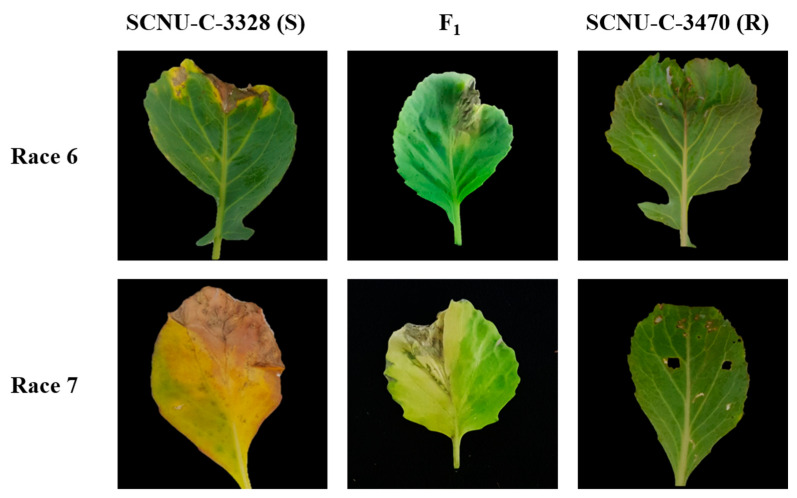
Phenotypes of the resistant and susceptible, and their F_1_ generation two weeks after inoculation with *Xanthomonas campestris* pv. *campestris* (*Xcc*) races 6 and 7.

We assessed the inheritance patterns of resistance to *Xcc* races 6 and 7 using an F_2_ population derived from a cross between the two cabbage lines ‘SCNU-C-3328’ and ‘SCNU-C-3470’, which differ in their resistance to black rot. F_1_ plants were all susceptible, suggesting that resistance to *Xcc* races 6 and 7 is inherited as a recessive trait (Figure 1). A total of 95 F_2_ plants were inoculated with *Xcc* race 6, resulting in 21 resistant plants and 72 plants developing symptoms. Excluding two dead plants, only 93 plants were used in this study (Figure S1A). Similarly, we inoculated total of 91 F_2_ plants with *Xcc* race 7, with 34 plants showing no symptoms and 55 plants developing a typical black rot disease. Only 89 plants were used in this study, excluding two dead plants (Figure S1B). A χ^2^ test revealed that resistance to *Xcc* race 6 follows a 3:1 (susceptible:resistant) segregation ratio, which is consistent with a single recessive gene conferring resistance (Table 1 and Figure S1). By contrast, F_2_ plants inoculated with race 7 showed a 1.6:1 (susceptible:resistant) segregation ratio, thus ruling out a status as a single monogenic trait.

### 2.2. Selection of Xcc Races 6 and 7 Resistance Gene

The identification of black-rot-resistant genotypes is a prerequisite for the development of cabbage cultivars resistant to black rot. In our previous work, we analyzed 157 NBS-LRR-encoding *R* genes, some of which were differentially expressed between black rot-resistant and -susceptible cabbage lines [17,20]. Expression levels of these *R* genes were determined by qRT-PCR (Figure S2). Only two genes, *Bol031422* and *Bol037412*, were more highly expressed in the resistant cabbage line SCNU-C-3470 than in the susceptible line SCNU-C-3328. Both *Bol031422* and *Bol037412* are intronless genes. *Bol031422* was highly expressed specifically in leaf, while *Bol037412* was highly expressed only in root, which is not a tissue affected by *Xcc* [17]. Taken together, these results suggested that *Bol031422* might play an important role in resistance to *Xcc* races 6 and 7 in cabbage.

### 2.3. Cloning and Sequencing of Candidate Gene

**Figure 2:**
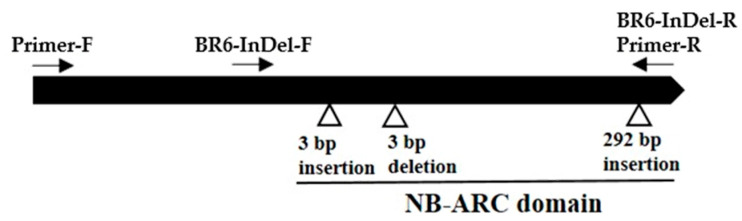
Primer location in *R* gene used in this study. ORF: Primer-F and Primer-F; Indel marker ‘BR6-InDel’: InDel-F and InDel-R.

**Figure 3:**
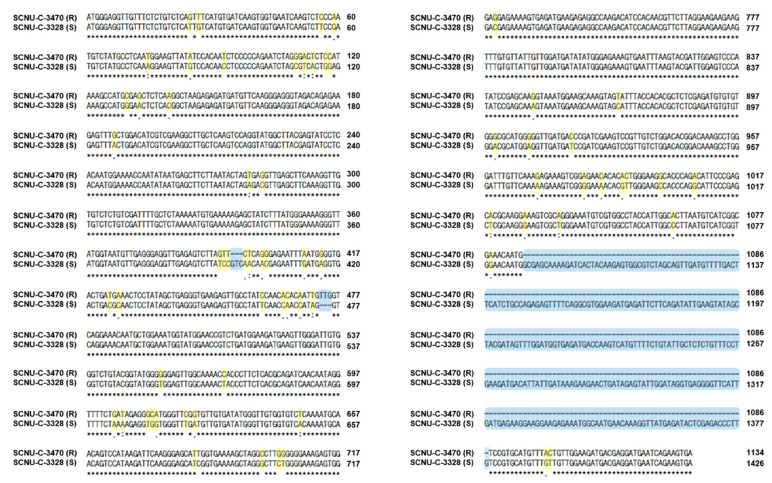
Sequence alignment between resistant (SCNU-C-3470, upper) and susceptible (SCNU-C-3328, lower) cabbage lines and position of InDel marker. The yellow and blue highlights in the alignment of DNA sequences indicate SNP and InDel, respectively.

**Figure 4:**
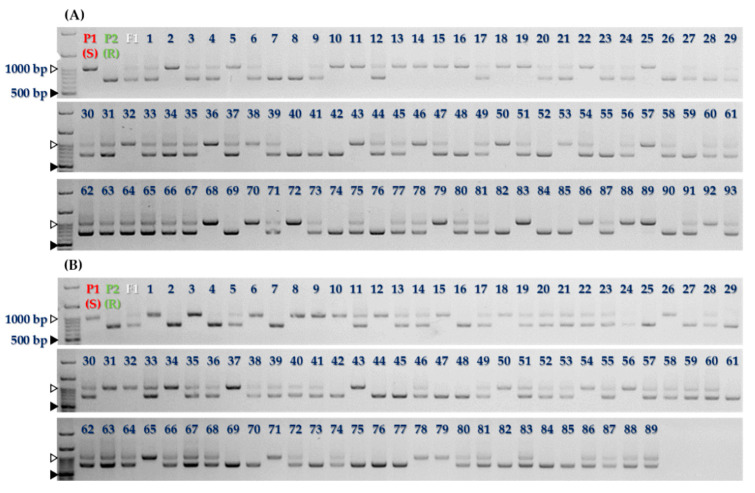
Banding pattern of InDel marker in resistant, susceptible lines (P1, SCNU-C-3328; P2, SCNU-C-3328), F1 and F2 generation. Inoculated with *Xcc* race 6 (**A**), *Xcc* race 7 (**B**). R, resistant; S, susceptible. Filled triangle; 1000 bp ladder, unfilled triangle; 500 bp ladder.

To detect sequence variation in the candidate *R* gene (*Bol031422*) for *Xcc* resistance, gene-specific primers were designed to cover the entire coding sequence (Figure 2 and Table S1). Sequencing of resistant and susceptible lines revealed multiple polymorphisms, consisting of several InDels and SNPs (Figure 3 and Figure S3A). Therefore, missense and non-synonymous mutations were found in the protein sequence (Figure S3B). The susceptible line SCNU-C-3328 showed a 3 bp insertion at nucleotide position 437, as well as a 3 bp deletion at nucleotide position 475. Accordingly, we designed primers to cover each three InDel regions and assess their correlation with *Xcc* resistance. Only one InDel segregated according to resistance across the resistant and susceptible lines (data not shown). This 292 bp insertion in the susceptible line SCNU-C-3328 at nucleotide position 1087 (Figure 2, Figure 3 and Figure S3A) resulted in a frameshift and premature translation termination (Figure S3B). We designed a new set of primers covering this InDel (Table 2), generating a PCR amplicon of 724 bp in resistant lines and 1013 bp in susceptible lines (Figure 4). The primers also amplified two bands from F_1_ plants derived from the cross between lines SCNU-C-3328 and SCNU-C-3470, as expected.

### 2.4. Validation of the InDel Marker

To test the association between the InDel marker BR6-InDel and resistance to black rot, we used two populations inoculated with *Xcc* races 6 or 7, respectively (Tables S2 and S3). The first population consisted of the F_2_ population described earlier, and the second population comprised 27 inbred cabbage lines. Genotyping analysis of the F_2_ population confirmed the close linkage of BR6-InDel with *Xcc* races 6 and 7 resistance, based on inoculation results (Figure 4 and Figure S4). Of the 27 inbred lines, two cabbage lines (‘SCNU-C-3470’ and ‘SCNU-C-4118’) were resistant to *Xcc* race 6, while four (‘SCNU-C-107’, ‘SCNU-C-3270’, ‘SCNU-C-3470’, and ‘SCNU-C-4059’) were resistant to race 7 (Table S3) [18]. A comparison of phenotypic results obtained from inoculation with *Xcc* races 6 or 7 and the genotype results with the InDel marker showed a predictive power of the InDel marker adaptability of 83.9% (83.5% in the F_2_ population; 85.2% in inbred lines) and 69.0% (67.0% in the F_2_ population; 76.0% in inbred lines), respectively. These results indicated that *Bol031422* might play an important role in resistance to *Xcc* race 6. In addition, the genotype at BR6-InDel appeared to better predict resistance against *Xcc* race 6 than to race 7.

## 3. Discussion

The aim of cabbage breeding is to increase yield and nutritional quality. However, diseases such as black rot, blackleg and clubroot cause severe yield loss in cabbage. Although chemical control is effective for combatting the disease, frequent use of bactericides is detrimental to the environment. In addition, crop pathogens are subjected to selective pressures, such as bactericides. Therefore, it is critical to develop disease-resistant cultivars via molecular breeding. The advancement of high-throughput sequencing technologies has allowed rapid implementation of molecular-marker-assisted selection to detect and utilize polymorphisms. Indeed, molecular markers have been instrumental in the selection of disease-resistant cultivars. *R* genes are high-confidence candidate genes for conferring resistance to a disease and can easily be turned into DNA markers. In this study, we developed the InDel marker BR6-InDel linked to black rot resistance against *Xcc* race 6.

Our research group recently reported 157 NBS-LRR-encoding *R* genes in *B. oleracea* [20], of which 32 were differentially expressed between leaves, roots, siliques, and stems [17]. Among these, only four genes (*Bol009890*, *Bol022619*, *Bol042095*, and *Bol031422*) were highly expressed in leaves. We also previously reported that cabbage line SCNU-C-3470 is resistant to *Xcc* races 6 and 7 [18]. Only two genes (*Bol031422* and *Bol037412*) of the 32 genes above were differentially expressed in SCNU-C-3470 compared to SCNU-C-3328, as determined by qRT-PCR. However, only *Bol031422* was highly expressed in leaves, while *Bol037412* was highly expressed in roots [17]. In addition, Basic Local Alignment Search Tool (BLAST) searches using the *Bol031422* sequence as query against the IRGSP-1.0, TAIR10, and Brapa_1.0 databases revealed sequence similarity to disease resistance genes, such as Os01g0788500 (disease resistance protein domain containing protein), At1g61180 (the LRR and NB-ARC domains containing disease resistance protein UNI), and Bra026923 (NBS-encoding gene in *B. rapa*).

In this study, the cabbage *R* gene *Bol031422*, located on chromosome C08, showed that it can be amplified from both resistant and susceptible lines and displays genotype-specific polymorphisms. Phenotyping of F_1_ and F_2_ cabbage plants derived from a cross between resistant and susceptible lines and their parents confirmed that the resistance trait segregates as a single recessive gene (Table 1). Indeed, all F_1_ plants were susceptible to infection by *Xcc* races 6 and 7. Furthermore, sequence analysis of *Bol031422* identified several InDels and SNPs in the susceptible line SCNU-C-3328 compared to the resistant line (Figure S3). In particular, a 292 bp insertion caused a frameshift and premature termination of translation was detected in SCNU-C-3328 (Figure 3), against which we designed the InDel marker BR6-InDel (Figure 2 and Table 2). The correlation between race 6 resistance phenotypes and the putative InDel marker genotyping results is only 83.9% (Table S2), suggesting *Bol031422* might be involved in black rot resistance against *Xcc* race 6. However, the genetic architecture of resistance to *Xcc* race 6 appears to be distinct from that against *Xcc* race 7, as F_2_ plants inoculated with race 7 showed a ratio of 1.6:1 (susceptible:resistant), and genotype results agreed with the phenotype for only 70% of plants. These results support the hypothesis that resistance to *Xcc* race 7 in cabbage relies on several genes, although *Bol031422* is likely to be a major contributor. More detailed studies of the gene responsible for races 6 and 7 resistance should be performed.

## 4. Materials and Methods

### 4.1. Plant Materials

The parental lines that were used in this study were ‘SCNU-C-3470’ and ‘SCNU-C-3328’, which are resistant and susceptible to *Xcc* races 6 and 7, respectively. ‘SCNU-C-3328’ was crossed with ‘SCNU-C-3470’ to develop the F_1_ generation. The F_2_ generation of 186 plants was developed via self-pollination of the F_1_ population. Of these F_2_ plants, 95 plants were used in *Xcc* race 6 and 91 plants were used in race 7. In addition, 27 cabbage inbred lines were also used in this study [18]. Cabbage seeds were sown in trays containing a nursery soil at plant culture room. Twenty-five days after sowing, the seedlings were transferred to pots. All plants were grown in a plant culture room at 24–27 °C under long-day conditions (14 h light/10 h dark cycles) with 60% relative humidity.

### 4.2. Bacterial Strains and Culture Media

*Xanthomonas campestris* pv. *campestris* (*Xcc*) races 6 and 7, which are causal agent of black rot of cabbage, were provided by the School of Life Sciences, University of Warwick, Coventry, UK (Table 3). Both *Xcc* strains were grown on petri dish containing 15 mL King’s B (KB) medium for 48 h at 30 °C [27].

### 4.3. Inoculation Test

Thirty-five days after sowing, the F_2_ plants and cabbage inbred lines were inoculated. The *Xcc* races were scraped from the culture plates and subcultured by suspending in liquid KB medium for 48 h at 30 °C. Thereafter, the bacterial suspension was adjusted to a concentration of 1.0 × 10^8^–10^9^ CFU/mL by adding sterile water. Finally, three youngest leaves of each plants were inoculated by mouse-tooth forceps methods at secondary veins (at least 10 inoculation sites per leaf). Then, inoculated plants were covered to maintain high relative humidity [9,28].

### 4.4. Disease Scoring

**Figure 5:**
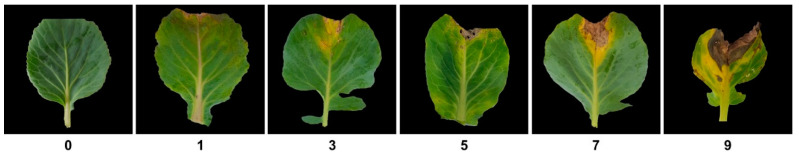
The disease rating criteria used in this study for black rot of cabbage. Scales are 0–9. 0, high resistant (HR); 1–3, resistant (R); 5–7, susceptible (S); 9, highly susceptible (HS).

To evaluate the disease symptoms of the inoculated leaves, the leaves at 2 weeks after inoculation (WAI) were used. The disease ratings were scored for each leaf at 2 WAI based on a 0–9 scale (Figure 5). Leaf symptoms were scored on the following scale: 0 = no visible symptoms, 1 = chlorosis or small necrosis near the inoculation site, 3 = typical small V-shaped lesion with black veins, 5 = typical lesion half way to the middle vein, 7 = typical lesion succeeding to the middle vein, and 9 = lesion reaching the middle vein as previously described [29]. Scales 0, 1–3, 5–7 and 9 were characterized as highly resistant (HR), resistant (R), susceptible (S) and highly susceptible (HS), respectively (Figure 5).

### 4.5. Isolation of Genomic DNA

Young leaf samples of each cabbage plants were collected and immediately frozen in liquid nitrogen. Genomic DNA was extracted using the DNeasy Plant Mini Kit (QIAGEN, Hilden, Germany) according to the manufacturer’s instructions. The purity and integrity of the DNA were assessed by ND-1000 Micro-spectrophotometer (NanoDrop Technologies Inc., Wilmington, DE, USA) and gel electrophoresis (0.8% agarose gel), respectively.

### 4.6. PCR Amplification

PCR amplification was done in 20 μL reaction mixture, comprising 10 μL of 2X Prime Taq premix (Genet Bio, Daejeon, Korea), 1.0 μL of each forward and reverse primers (10 pmoles), 2 μL of 100 ng genomic DNA as template and 6 μL of deionized distilled water. Primers are listed in Table 2 and Table S1. The PCR conditions were as follows: 95 °C for 5 min, followed by amplifications of 30–35 cycles at 95 °C for 30 s, 58 and 60 °C for 30 s (specific Tm to respective primer sets in Table 2 and Table S1), 72 °C for 30 s and 72 °C for 5 min. Then, amplified PCR products were assessed by gel electrophoresis (1.5% agarose gel).

### 4.7. Cloning and Sequencing

The candidate *R* gene (*Bol031422*) was amplified by Phusion High-Fidelity DNA Polymerase (New England Biolabs, EVRY Cedex, France) from the genomic DNA of *Xcc* resistant and susceptible cabbage lines. Amplified DNA fragments were extracted from the gel and purified using the Wizard SV gel and PCR cleanup system (Promega, Madison, WI, USA). Then, gene cloning was performed using the TOPO TA cloning kit (Invitrogen, Carlsbad, CA, USA). The cloned amplicons were sequenced with the universal primers, M13F and M13RpUC, using ABI 3730XL DNA sequencer (Macrogen Co., Seoul, Korea). To remove all ambiguities, each forward and reverse sequence of resistant and susceptible cabbage lines was repeated five times. Gene sequences between the resistant and susceptible lines were compared using the multiple sequence alignment by CLUSTALW (https://www.genome.jp/tools-bin/clustalw, accessed on 4 March 2021).

### 4.8. RNA Extraction and qRT-PCR Analysis

Total RNAs were extracted from 100 mg of finely ground leaf tissue at 35 days after sowing using RNeasy mini kit (QIAGEN, Hilden, Germany). Total RNA concentration and quality were measured using a ND-1000 Micro-spectrophotometer. SuperScript^®^ III First-Strand Synthesis System kit (Invitrogen, Gaithersburg, MD, USA) was used for cDNA synthesis from total RNA. The cDNA was then used for real-time quantitative PCR with the LightCycler system (Roche, Mannheim, Germany) instrument using qPCRBIO SyGreen Mix Lo-ROX (PCR Biosystems, London, UK) for NBS-encoding genes. Primers were listed in Afrin et al. [17]. Threshold cycle (Ct) values were used to calculate 2^−ΔΔCT^ method, with actin used as an internal control [30].

### 4.9. Statistical Analysis

A Chi-square (χ^2^) test for goodness-of-fit was performed to determine deviations of observed data from the expected segregation ratios using XLSTAT software. Data are presented as mean ± standard error of the mean. Statistical differences were assessed by Student’s *t*-test. The significance of differences between the means was assessed using *p*-values of <0.01 and <0.05. All the statistical analyses were performed using PRISM 6 software (ver. 6.01, GraphPad Software Inc., San Diego, CA, USA) [31].

## 5. Conclusions

This study showed that *Xcc* race 6 resistance in cabbage line ‘SCNU-C-3470’ is regulated by a single recessive gene. The newly developed InDel marker ‘BR6-InDel’ is linked to *Xcc* races 6 and 7 resistance and located on cabbage chromosome C08, showed consistency with phenotypic results of both the inbred cabbage lines and F_2_ population. This InDel marker could be valuable for future cabbage breeding programs.

## Figures and Tables

**Table 1:** Inheritance of *Xcc* races 6 and 7 resistance in cabbage. S; susceptible, R; resistant.

Crosses	Generation	Susceptible	Resistant	Expected Ratio (S:R)	Chi-Square (χ^2^)	*p*
Race 6						
SCNU-C-3328 (S)	P_1_	12	0			
SCNU-C-3470 (R)	P_2_	0	12			
3328 × 3470	F_1_	12	0			
3328 × 3470	F_2_	72	21	3:1	0.29	0.59
Race 7						
SCNU-C-3328 (S)	P_1_	12	0			
SCNU-C-3470 (R)	P_2_	0	12			
3328 × 3470	F_1_	12	0			
3328 × 3470	F_2_	55	34	3:1	8.27	0.004

**Table 2:** Primer specifications of InDel marker associated with *Xcc* resistance in cabbage.

Gene ID	InDel Marker		Primer (5′-3′)	Tm (°C)	Product Size
*Bol031422*	BR6-InDel	F	TGGGGTGACTGATGAAACTCCTAT	60	724 bp
		R	TCACTTCTGATTCATCCTCGTCATCT		

**Table 3:** *Xanthomonas campestris* pv. *campestris* (*Xcc*) races used in this study.

Sl. No.	Bacterial Race/Strains	Source	Reference
1	*Xanthomonas campestris* pv. *campestris* Race 6 (6181)	Portugal	[9]
2	*Xanthomonas campestris* pv. *campestris* Race 7 (8450A)	UK	

## Data Availability

Not applicable.

## Supplementary Materials

The following are available online at www.mdpi.com/article/10.3390/plants10091940/s1, Figure S1. Disease symptoms of cabbage F2populations 14 days after being inoculation with *Xanthomonascampestris* pv. *campestrisraces* 6 (**A**) and 7 (**B**).; Figure S2. Mean normalized expression of candidate genes in susceptible (SCNU-C-3328) and resistant (SCNU-C-3470) cabbage lines. Error bar represents ± S.E.M of the means of triplicates. Asterisks indicate significant difference in expression level (* *p* < 0.05, ** *p* < 0.01, Student’s *t*-test).; Figure S3. Sequence information obtained by cloning and sequencing of cabbage resistant (SCNU-C-3470) and susceptible (SCNU-C-3328) lines for a NBS-LRR gene (Bol031422) and their alignments. Genomic DNA (gDNA) sequence and their alignment (A), protein sequence and their alignment (B). Sequence alignments along with the references sequences retrieved from Bolbase (http://www.ocri-genomics.org/bolbase/) considering Brassica oleracea as reference genome database. In sequence alignment asterisks (*) indicate sequence similarity. Absence of asterisks indicates sequence dissimilarity. En dash (–) indicates insertion/deletion of nucleotides. The grey and red highlights in the alignment of genomic DNA sequences indicate the BR6-InDel-F and BR6-InDel-R, respectively. The red text colors in the alignment of genomic sequences indicate InDel regions.; Figure S4. Banding profile of R gene (*Bol031422*) in 27 inbred cabbage lines using Pimer-F/R (**A**), BR6-InDel-F/R (**B**).; Table S1. Primer specifications of candidate Black rot resistance NBS gene *Bol031422*.; Table S2. Phenotypic and Genotypic screening results for against *Xcc* race 6 and 7 in cabbage. S; susceptible, H; Hetero, R; Resistant.; Table S3. Comparison of bioassay and molecular screening results for black rot resistance in cabbage inbred lines. S; susceptible, H; hetero, R; resistant.
