# Supplementary material for: Inheritance of Black Rot Resistance and Development of Molecular Marker Linked to Xcc Races 6 and 7 Resistance in Cabbage
Source: Plants (Basel). 2021 Sep 17;10(9):1940. doi: 10.3390/plants10091940 (PMC8472523; doi:10.3390/plants10091940)
Supplement: Supplementary file 1 [file plants-10-01940-s001.zip › Figure S1.pdf]

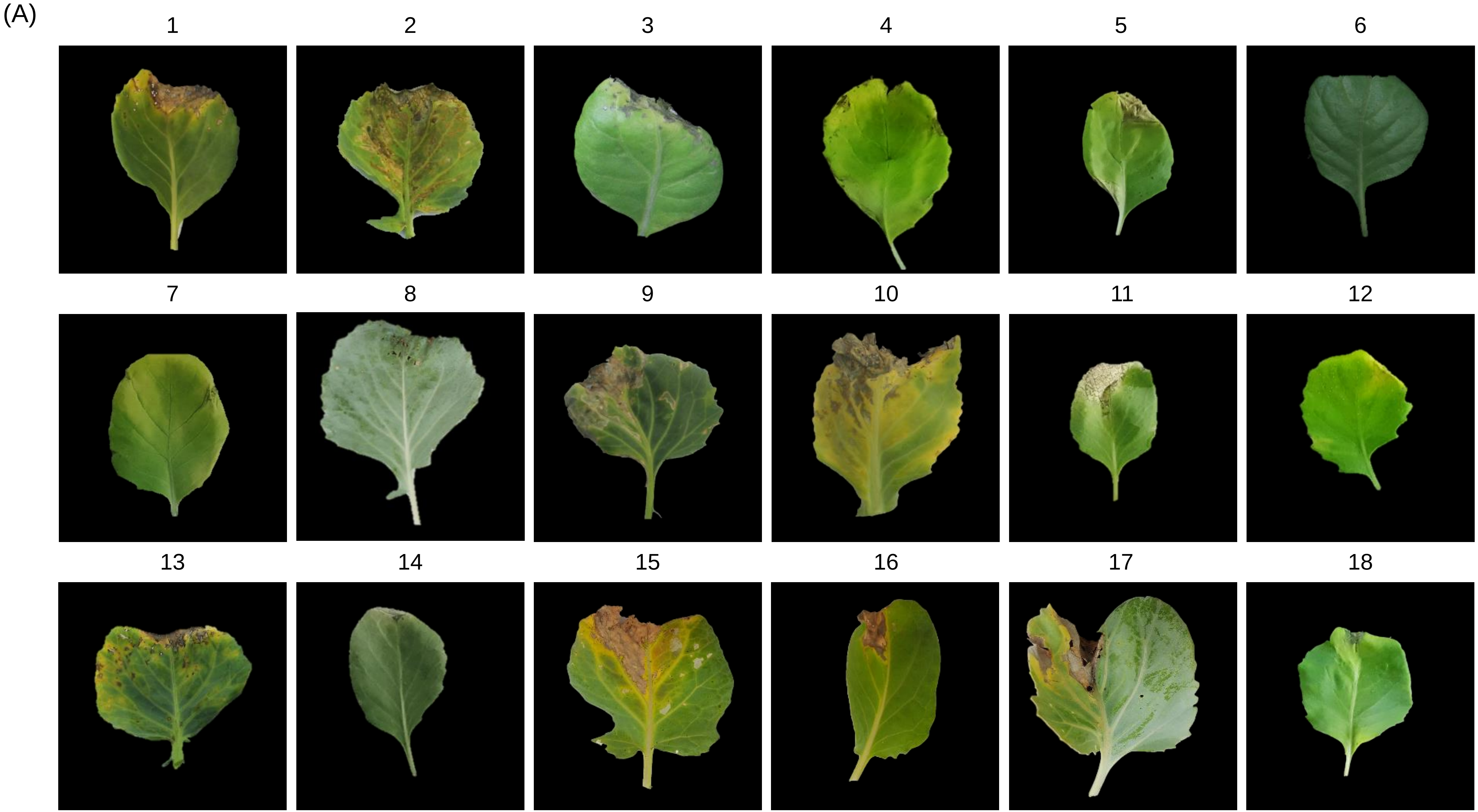
(A)
1
2
3
4
5
6
7
8
9
10
11
12
13
14
15
16
17
18

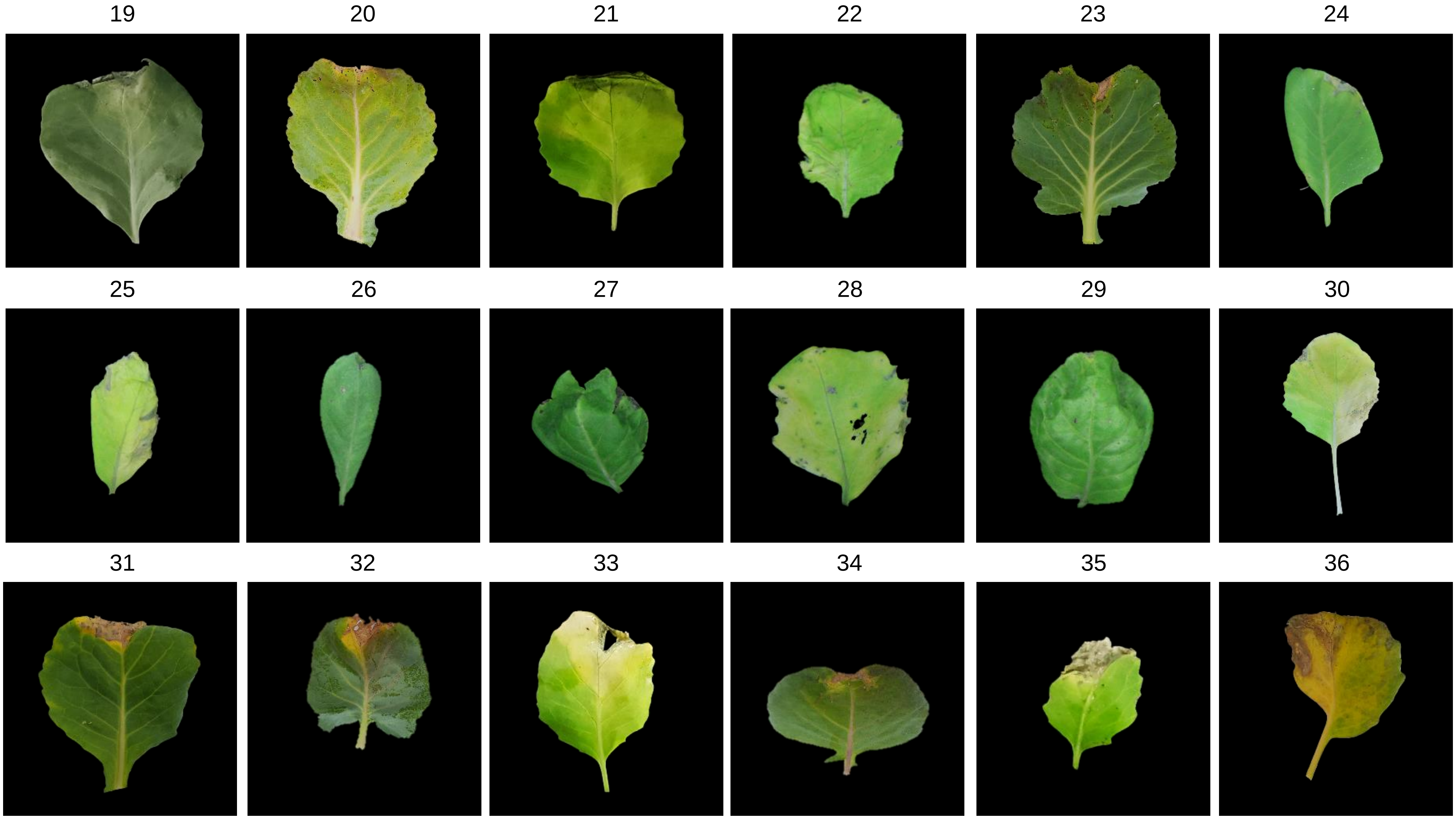
19
20
21
22
23
24
25
26
27
28
29
30
31
32
33
34
35
36

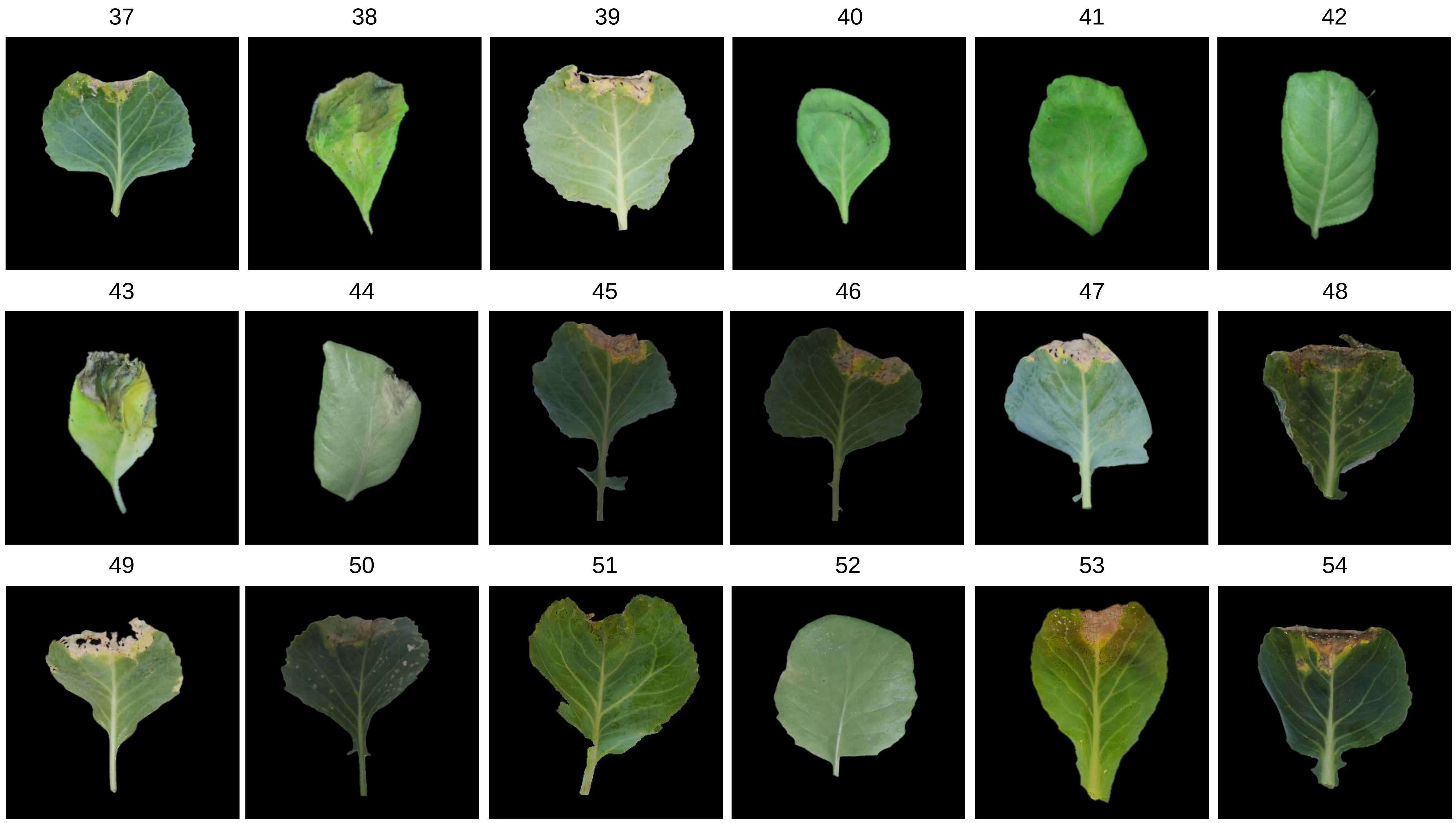
37
38
39
40
41
42
43
44
45
46
47
48
49
50
51
52
53
54

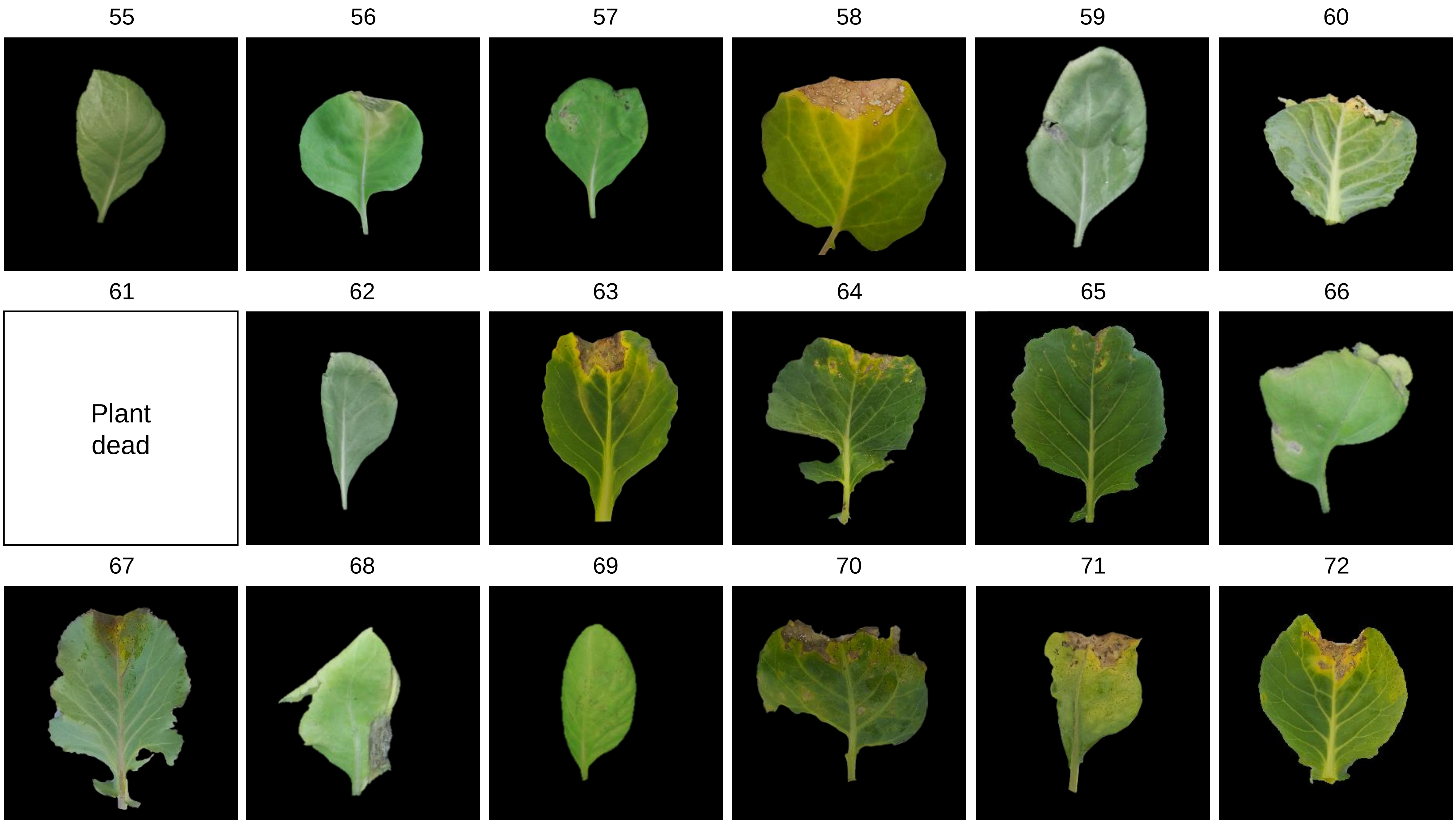
55
56
57
58
59
60
61
Plant dead
62
63
64
65
66
67
68
69
70
71
72

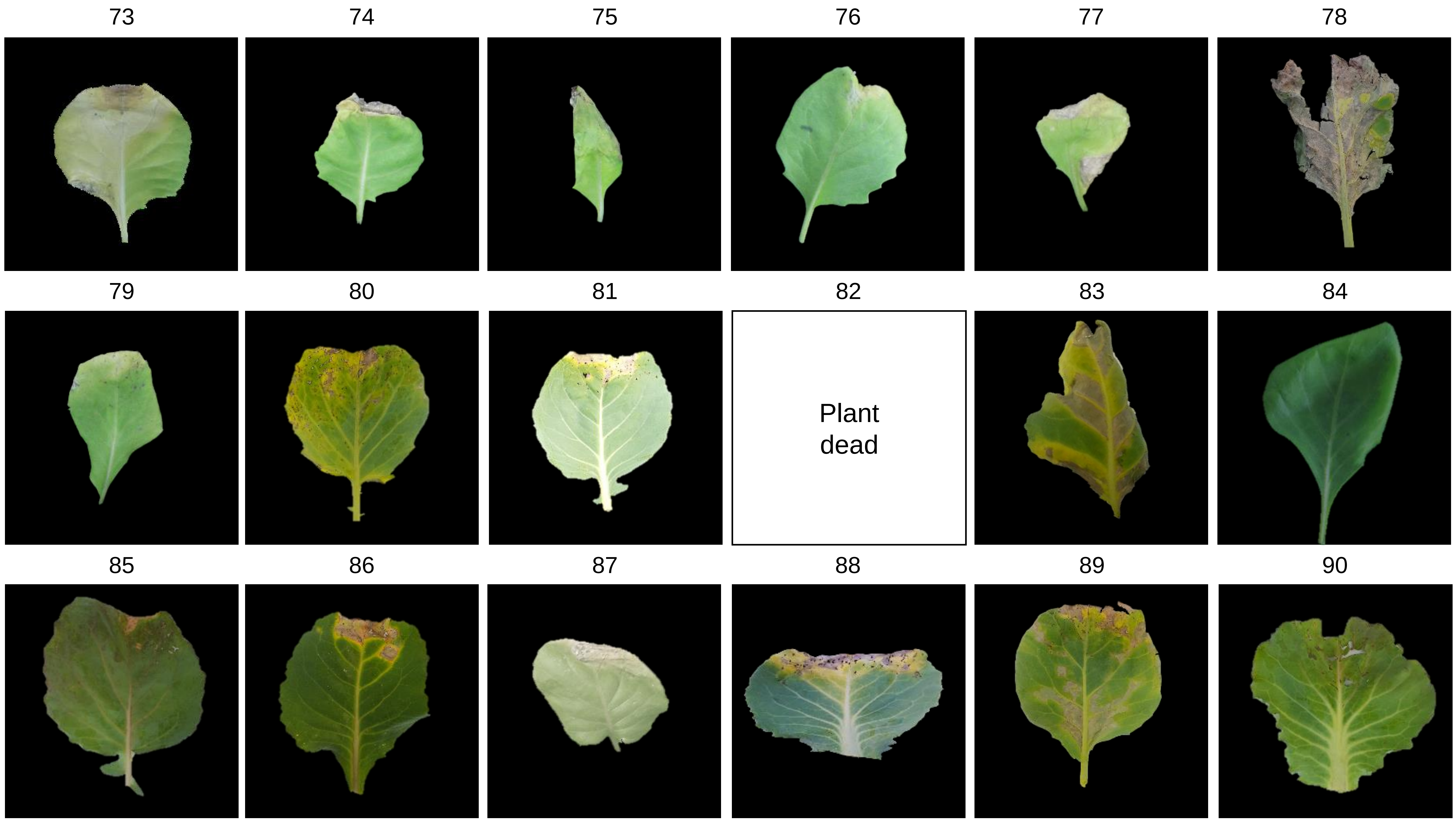
73
74
75
76
77
78
79
80
81
82
Plant dead
83
84
85
86
87
88
89
90

91

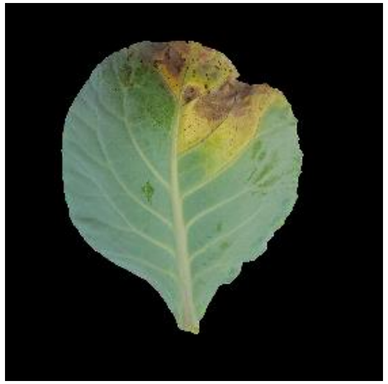

92

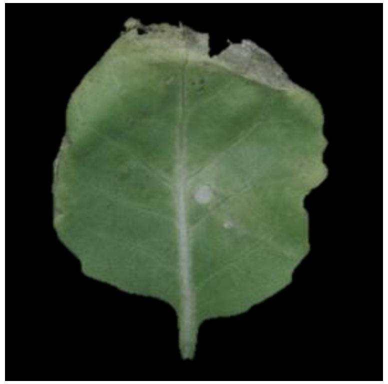

93

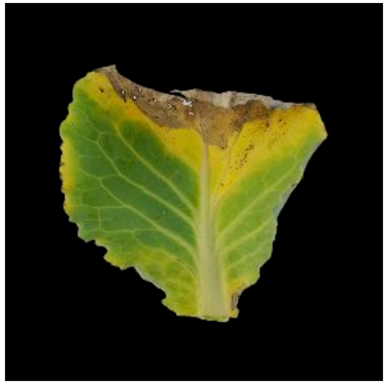

94

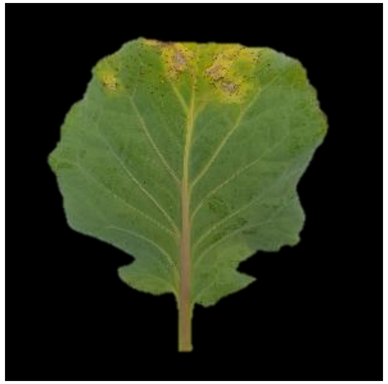

95

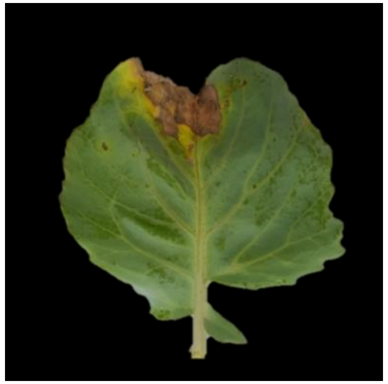

(B)

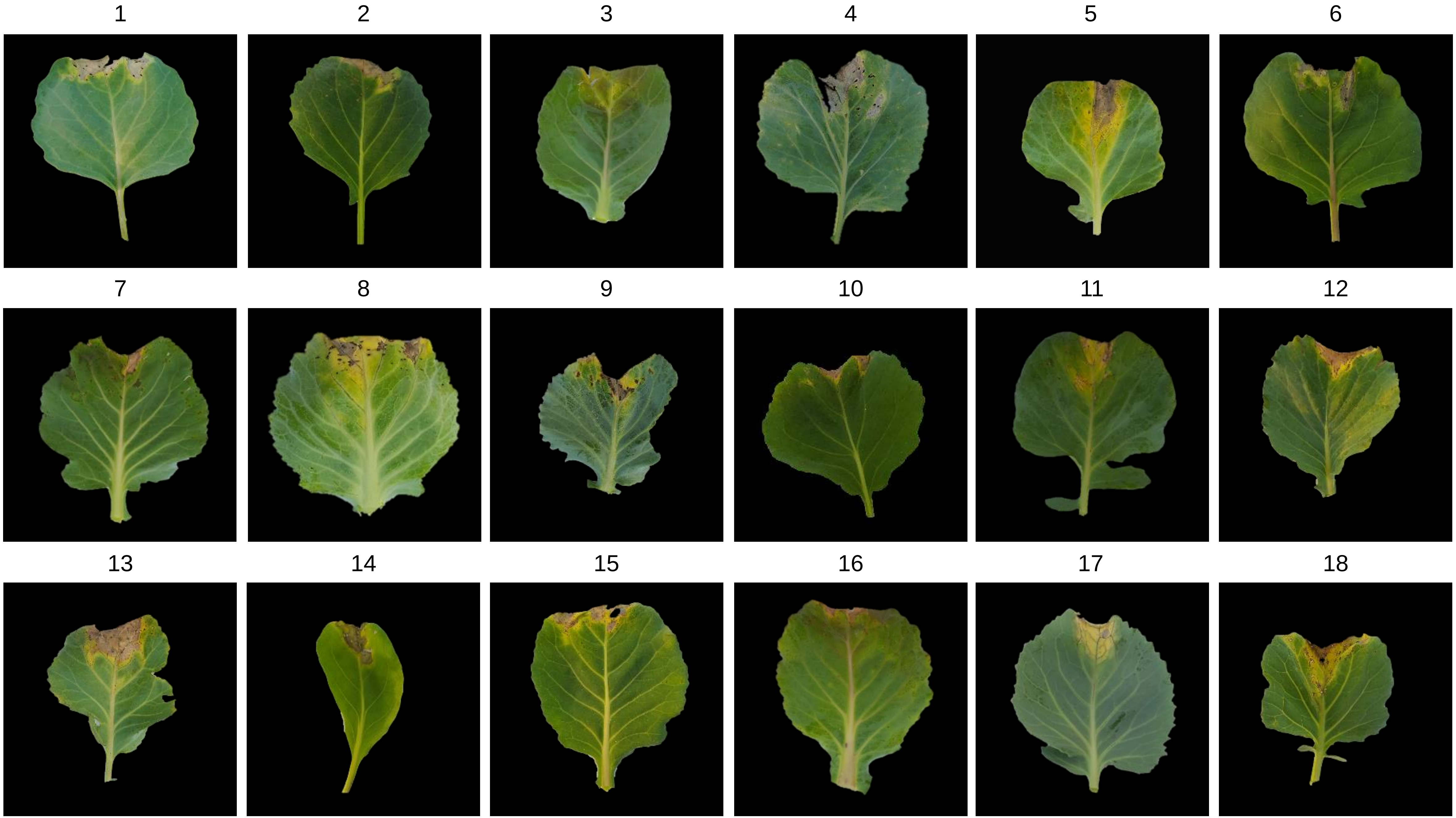

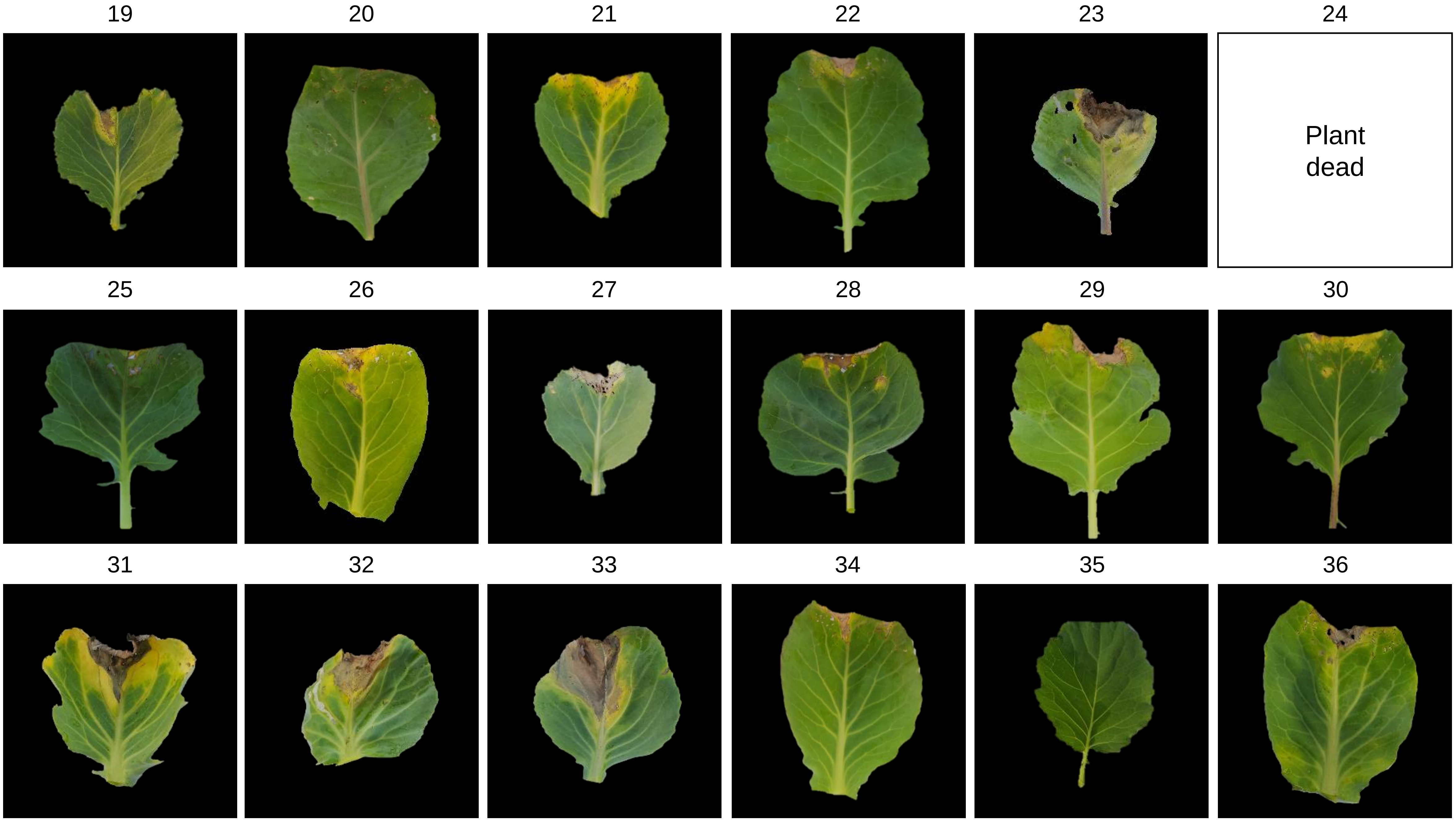
19
20
21
22
23
24
Plant dead
25
26
27
28
29
30
31
32
33
34
35
36

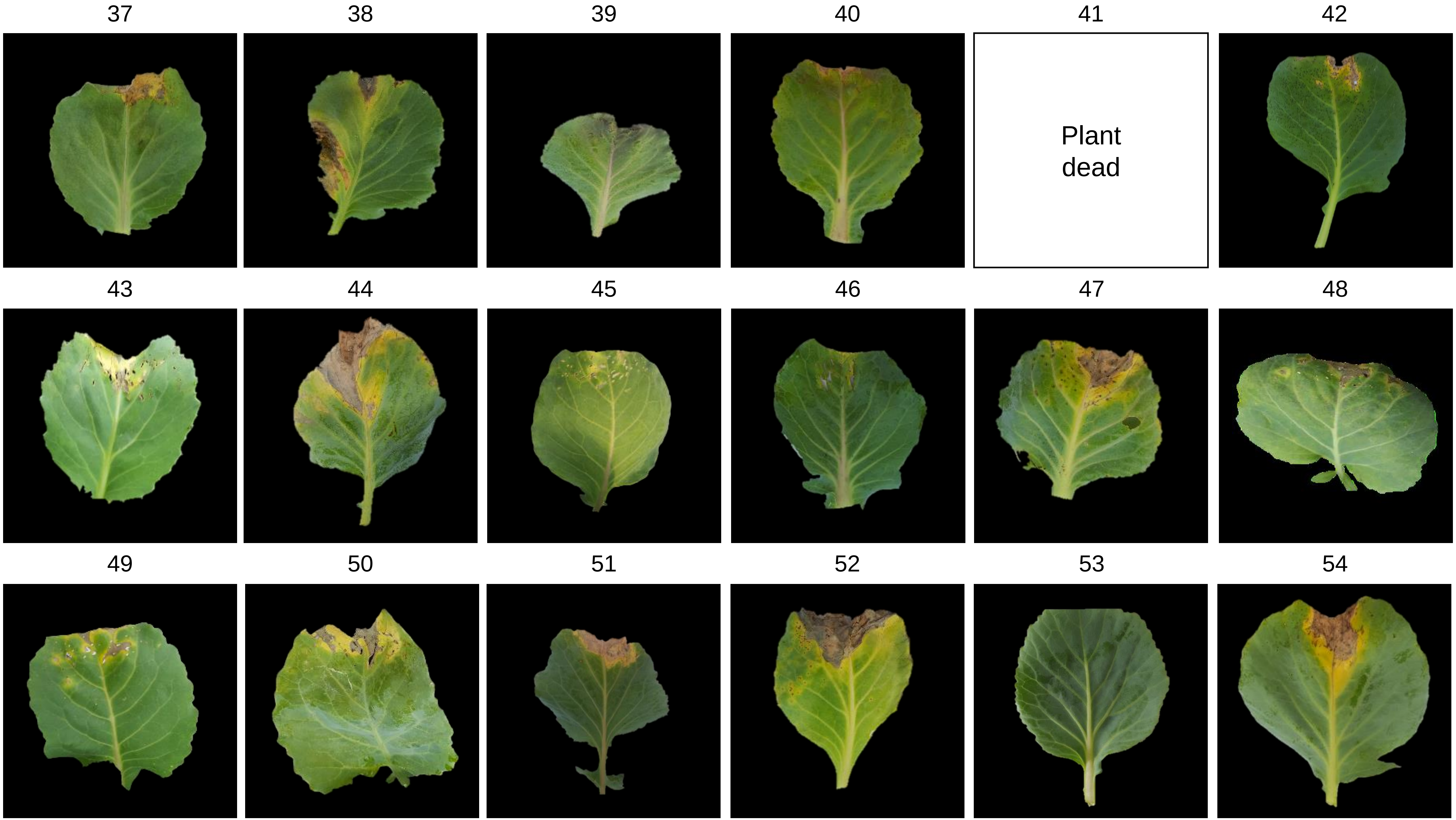
37
38
39
40
41
42
Plant dead
43
44
45
46
47
48
49
50
51
52
53
54

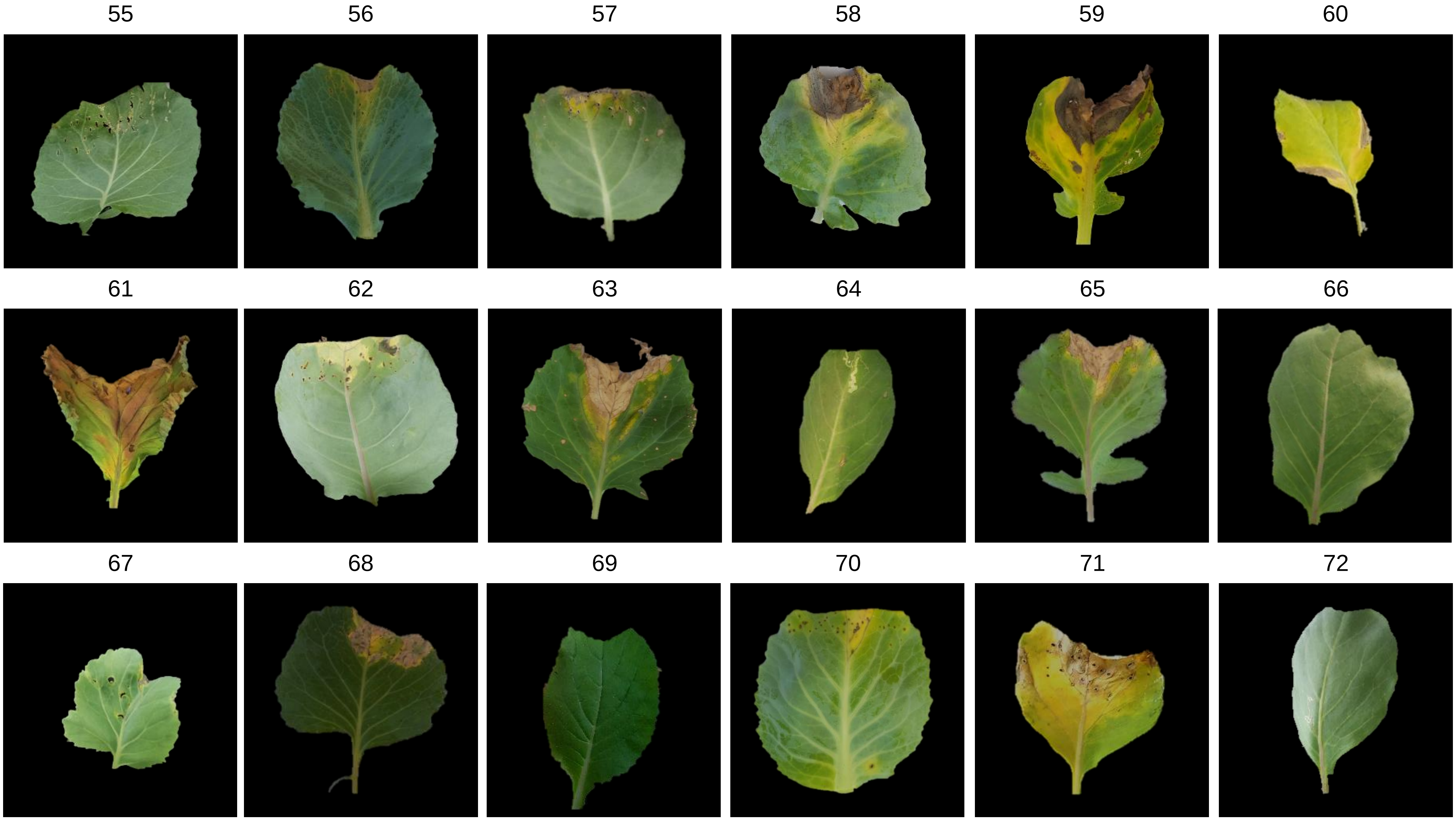
55
56
57
58
59
60
61
62
63
64
65
66
67
68
69
70
71
72

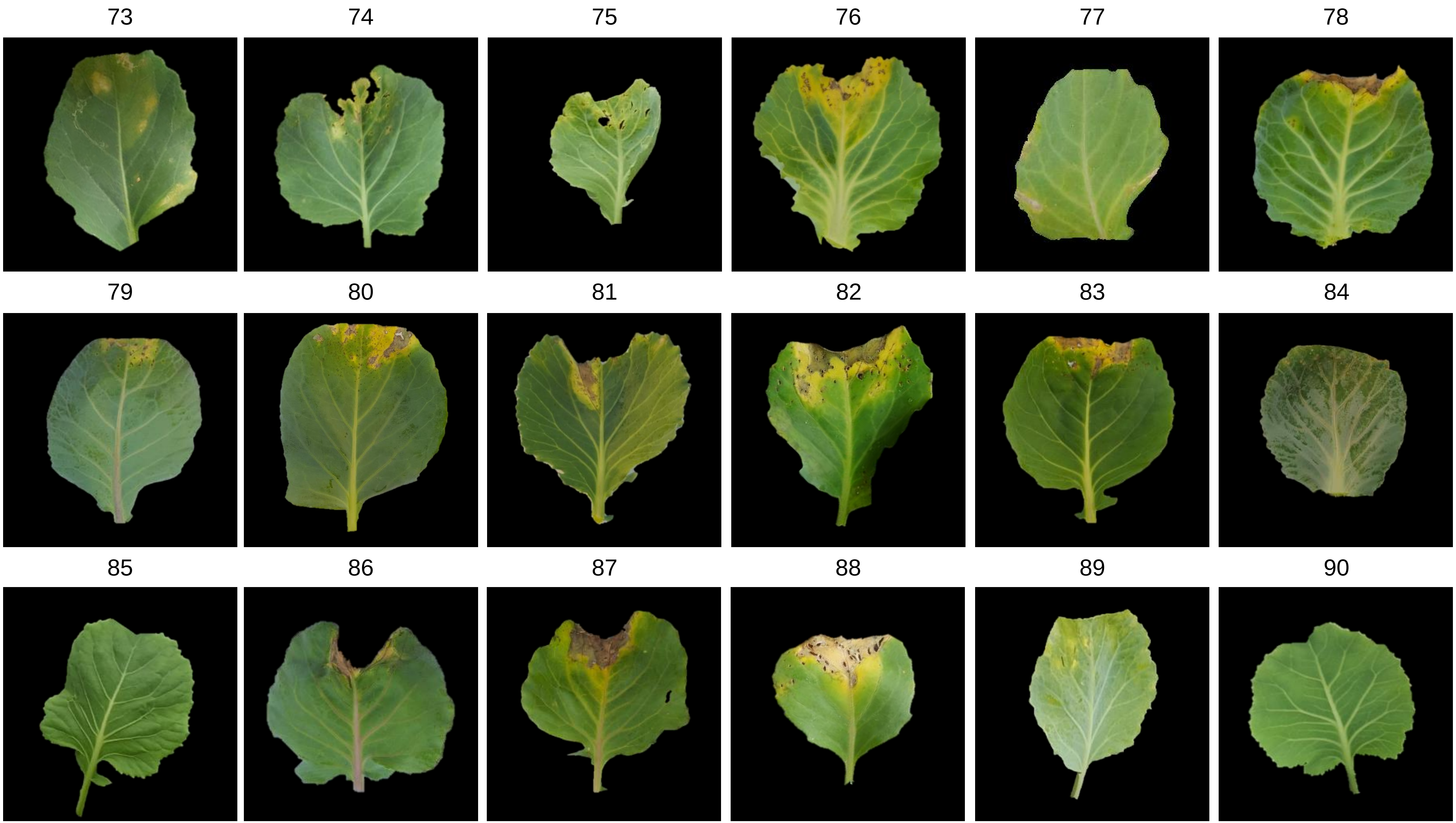
73
74
75
76
77
78
79
80
81
82
83
84
85
86
87
88
89
90

91

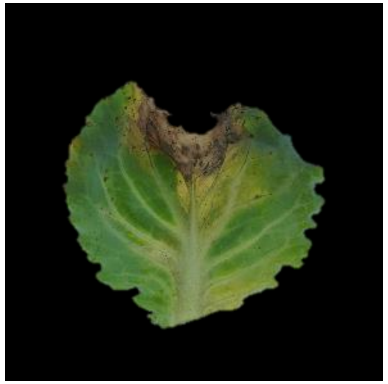

**Figure S1**. Disease symptoms of cabbage $F_2$ populations 14 days after being inoculation with *Xanthomonas campestris* pv. *campestris* races 6 (A) and 7 (B).
