# Supplementary material for: Inheritance of Black Rot Resistance and Development of Molecular Marker Linked to Xcc Races 6 and 7 Resistance in Cabbage
Source: Plants (Basel). 2021 Sep 17;10(9):1940. doi: 10.3390/plants10091940 (PMC8472523; doi:10.3390/plants10091940)
Supplement: Supplementary file 1 [file plants-10-01940-s001.zip › Figure S2.pdf]

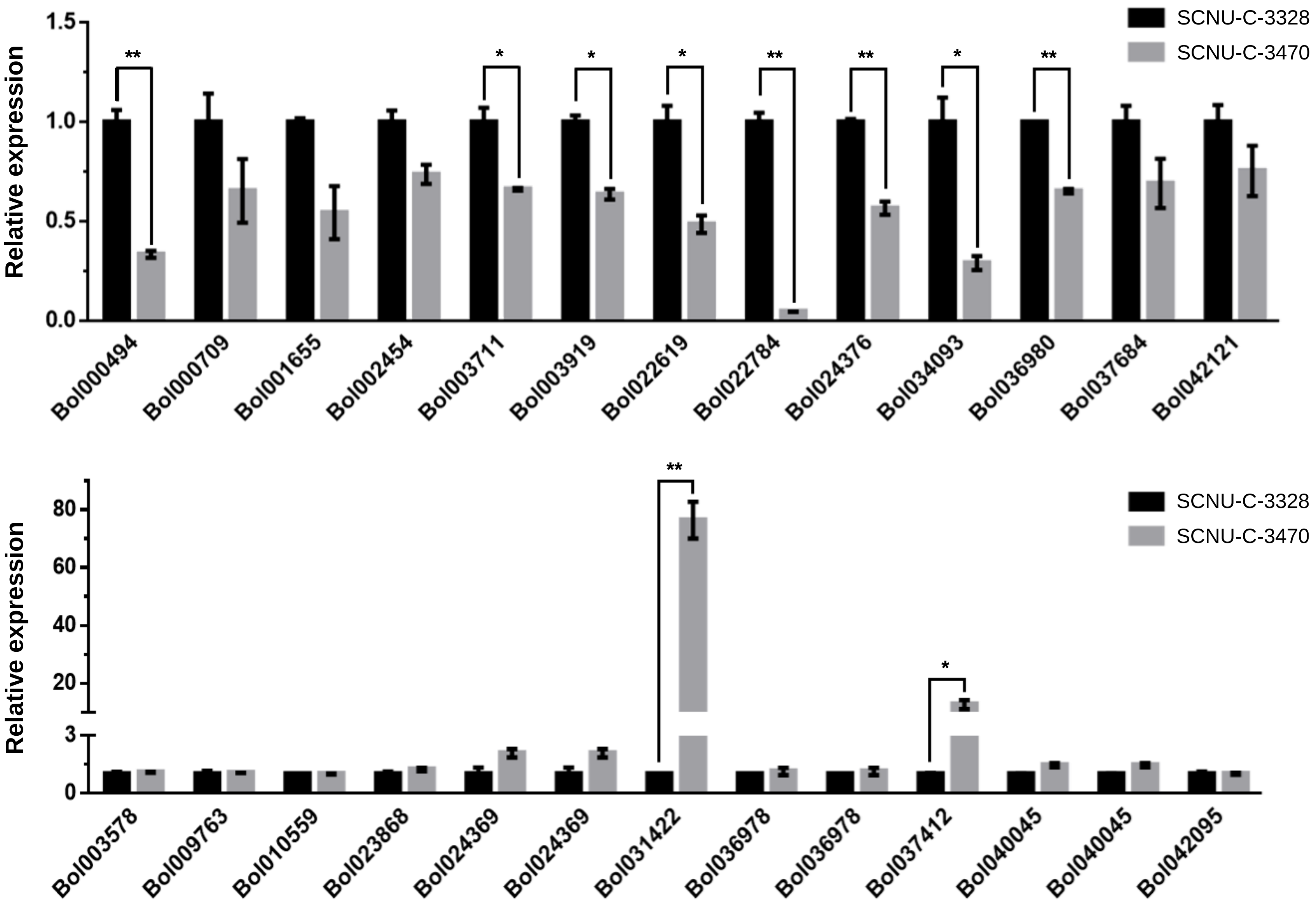


**Figure S2**. Mean normalized expression of candidate genes in susceptible (SCNU-C-3328) and resistant (SCNU-C-3470) cabbage lines. Error bar represents ± S.E.M of the means of triplicates. Asterisks indicate significant difference in expression level (*P < 0.05, **P < 0.01, Student's t-test).
