# Supplementary material for: Inheritance of Black Rot Resistance and Development of Molecular Marker Linked to Xcc Races 6 and 7 Resistance in Cabbage
Source: Plants (Basel). 2021 Sep 17;10(9):1940. doi: 10.3390/plants10091940 (PMC8472523; doi:10.3390/plants10091940)
Supplement: Supplementary file 1 [file plants-10-01940-s001.zip › Figure S3.pdf]

(A)

- Genomic DNA (gDNA) sequence

>Bol031422 (gDNA) Reference
ATGGGAGGTTGTTTCTCTGTCTCAGTTTCATGTGATCAAGTGGTGAATCAAGT
CTCCCAATGTCTATGCCTCAATGGAAGTTATATCCACAATCTCCCCCAGAATCT
AGGGACTCTCCATAAAGCCATGCGAGCTCTCAAGGCTAAGAGAGATGATGTT
CAAGGGAGGGTAGACAGAGAAGAGTTTGCTGGACATCGTCGAAGGCTTGCT
CAAGTCCAGGTATGGCTTACGAGTATCCTCACAATGGAAAACCAATATAATGA
GCTTCTTAATACTAGTGAGGTTGAGCTTCAAAGGTTGTGTCTCTGTCGATTTTG
CTCTAAAAATGTGAAAAAGAGCTATCTTTATGGGAAAAGGGTTATGGTAATGT
TGAGGGAGGTTGAGAGTCTTAGTTCTCAGGGAGAATTTAATGGGGTGACTGA
TGAAACTCCTATAGCTGAGGGTGAAGAGTTGCCTATTCAACCAACCATAGGTC
AGGAAACAATGCTGGAAATGGTATGGAACCGTCTGATGGAAGATGAAGTTGG
GATTGTGGGTCTGTACGGTATGGGGGGAGTTGGCAAAACCACCCTTCTCACG
CAGATCAACAATAGGTTTTCTGATAGAGGGCATGGGTTCGGTGTTGTGATATG
GGTTGTGGTGTCTCAAAATGCAACAGTCCATAAGATTCAAGGGAGCATTGGT
GAAAAGCTAGGCCTTGGGGGGAAAGAGTGGGAGGAGAAAAGAGAGATGAA
GAGAGGCCAAGACATCCACAACGTTCTTAGGAAGAAGAAGGTTGTGTTATTG
TTGGATGATATATGGGAGAAAGTGAATTTAAGTACGATTGGAGTCCCATATCC
GAGCAAGGTAAATGGAAGCAAAGTAGTATTTACCACACGCTCTCGAGATGTG
TGTGGGCGCATGGGGGTTGATGACCCGATCGAAGTCCGTTGTCTGGACACGG
ACAAAGCCTGGGATTTGTTCAAAGAGAAAGTCGGAGAACACACACTGGGAA
GGCACCCAGACATTCCCGAGCACGCAAGGAAAGTCGCAGGGAAATGTCGTG
GCCTACCATTGGCACTTAATGTCATCGGCGAAACAATGTCCGTGCATGTTTAC
TGTTGGAAGATGACGAGGATGAATCAGAAGTGA

>SCNU-C-3470 (gDNA) Resistant line
ATGGGAGGTTGTTTCTCTGTCTCAGTTTCATGTGATCAAGTGGTGAATCAAGT
CTCCCAATGTCTATGCCTCAATGGAAGTTATATCCACAATCTCCCCCAGAATCT
AGGGACTCTCCATAAAGCCATGCGAGCTCTCAAGGCTAAGAGAGATGATGTT
CAAGGGAGGGTAGACAGAGAAGAGTTTGCTGGACATCGTCGAAGGCTTGCT
CAAGTCCAGGTATGGCTTACGAGTATCCTCACAATGGAAAACCAATATAATGA
GCTTCTTAATACTAGTGAGGTTGAGCTTCAAAGGTTGTGTCTCTGTCGATTTTG
CTCTAAAAATGTGAAAAAGAGCTATCTTTATGGGAAAAGGGTTATGGTAATGT

TGAGGGAGGTTGAGAGTCTTAGTTCTCAGGGAGAATTTAATGGGGTGACTGA
TGAAACTCCTATAGCTGAGGGTGAAGAGTTGCCTATCCAACACACAATTGTTG
GTCAGGAAACAATGCTGGAAATGGTATGGAACCGTCTGATGGAAGATGAAGT
TGGGATTGTGGGTCTGTACGGTATGGGGGGAGTTGGCAAAACCACCCTTCTC
ACGCAGATCAACAATAGGTTTTCTGATAGAGGGCATGGGTTCGGTGTTGTGAT
ATGGGTTGTGGTGTCTCAAAATGCAACAGTCCATAAGATTCAAGGGAGCATT
GGTGAAAAGCTAGGCCTTGGGGGGAAAGAGTGGGAGGAGAAAAGTGAGAT
GAAGAGAGGCCAAGACATCCACAACGTTCTTAGGAAGAAGAAGTTTGTGTT
ATTGTTGGATGATATATGGGAGAAAGTGAATTTAAGTACGATTGGAGTCCCAT
ATCCGAGCAAGGTAAATGGAAGCAAAGTAGTATTTACCACACGCTCTCGAGA
TGTGTGTGGGCGCATGGGGGTTGATGACCCGATCGAAGTCCGTTGTCTGGAC
ACGGACAAAGCCTGGGATTTGTTCAAAGAGAAAGTCGGAGAACACACACTG
GGAAGGCACCCAGACATTCCCGAGCACGCAAGGAAAGTCGCAGGGAAATGT
CGTGGCCTACCATTGGCACTTAATGTCATCGGCGAAACAATGTCCGTGCATGT
TTACTGTTGGAAGATGACGAGGATGAATCAGAAGTGA

>SCNU-C-3328 (gDNA) Susceptible line
ATGGGAGGTTGTTTCTCTGTCTCATTGTCATGTGATCAAGTGGTGAATCAAGTC
TTCCGATGTCTATGCCTCAAAGGAAGTTATGTCCACAACCTCCCCCAGAATCT
AGCGTCACTGGAGAAAGCCATGGGAACTCTCACGGCTAAGAGAGATGATGTT
CAAGGGAGGGTAGACAGAGAAGAGTTTACTGGACATCGTCGAAGGCTTGCT
CAAGTCCAGGTATGGCTTACGAGTATCCTCACAATGGAAAACCAATATAATGA
GCTTCTTAATACTAGAGACGTTGAGCTTCAAAGGTTGTGTCTCTGTCGATTTTG
CTCTAAAAATGTGAAAAAGAGCTATCTTTATGGGAAAAGGGTTATGGTAATGT
TGAGGGAGGTTGAGAGTCTTATCCGTCAACAACGAGAATTTGATGAGGTGAC
TGACGCAACTCCTATAGCTGAGGGTGAAGAGTTGCCTATTCAACCAACCATA
GGTCAGGAAACAATGCTGGAAATGGTATGGAACCGTCTGATGGAAGATGAA
GTTGGGATTGTGGGTCTGTACGGTATGGGTGGAGTTGGCAAAACTACCCTTCT
CACGCAGATCAACAATAGGTTTTCTAAAAGAGGTGGTGGGTTTGATGTTGTGA
TATGGGTTGTGGTGTCACAAAATGCAACAGTCCATAAGATTCAAGGGAGCAT
CGGTGAAAAGCTAGGGCTTCTGGGGAAAGAGTGGGACGAGAAAAGTGAGAT
GAAGAGAGGCCAAGACATCCACAACGTTCTTAGGAAGAAGAAGTTTGTGTT
ATTGTTGGATGATATATGGGAGAAAGTGAATTTAAGTACGATTGGAGTCCCAT
ATCCGAGCAAAGTAAATGGAAGCAAAGTAGCATTTACCACACGCTCTCGAGA
TGTGTGTGGACGCATGGAGGTTGATGATCCGATCGAAGTCCGTTGTCTGGACA
CGGACAAAGCCTGGGATTTGTTCAAAAAGAAAGTCGGGGAAAACACGTTGG

GAAGCCACCCAGGCATTCCCGAGCTCGCAAGGGAAGTCGCTGGGAAATGTC
GTGGCCTACCATTGGCCCTTAATGTCATCGGTGGAACAATGGCGAGCAAAAG
ATCACTACAAGAGTGGCGTCTAGCAGTTGATGTTTTGACTTCATCTGCCAGAG
AGTTTTCAGGCGTGGAAGATGAGATTCTTCAGATATTGAAGTATAGCTACGAT
AGTTTGGATGGTGAGATGACCAAGTCATGTTTTCTGTATTGCTCTCTGTTTCCT
GAAGATGACATTATTGATAAAGAAGAACTGATAGAGTATTGGATAGGTGAGG
GGTTCATTGATGAGAAGGAAGGAAGAGAAATGGCAATGAACAAAGGTTATG
AGATACTCGAGACCCTTGTCCGTGCATGTTTGTTGTTGGAAGATGACGAGGAT
GAATCAGAAGTGA

- gDNA alignment

```
Bol031422
ATGGGAGGTTGTTTCTCTGTCTCAGTTTCATGTGATCAAGTGGTGAATCAAGTCTCCCAA    60
SCNU-C-3470
ATGGGAGGTTGTTTCTCTGTCTCAGTTTCATGTGATCAAGTGGTGAATCAAGTCTCCCAA    60
SCNU-C-3328
ATGGGAGGTTGTTTCTCTGTCTCATTGTCATGTGATCAAGTGGTGAATCAAGTCTTCCGA    60
************************ * **************************** **.*

Bol031422
TGTCTATGCCTCAATGGAAGTTATATCCACAATCTCCCCCAGAATCTAGGGACTCTCCAT    120
SCNU-C-3470
TGTCTATGCCTCAATGGAAGTTATATCCACAATCTCCCCCAGAATCTAGGGACTCTCCAT    120
SCNU-C-3328
TGTCTATGCCTCAAAGGAAGTTATGTCCACAACCTCCCCCAGAATCTAGCGTCACTGGAG    120
**************.*********.******* *************** *.*.**   *

Bol031422
AAAGCCATGCGAGCTCTCAAGGCTAAGAGAGATGATGTTCAAGGGAGGGTAGACAGAGAA    180
SCNU-C-3470
AAAGCCATGCGAGCTCTCAAGGCTAAGAGAGATGATGTTCAAGGGAGGGTAGACAGAGAA    180
SCNU-C-3328
AAAGCCATGGGAACTCTCACGGCTAAGAGAGATGATGTTCAAGGGAGGGTAGACAGAGAA    180
********* **.******.****************************************

Bol031422
GAGTTTGCTGGACATCGTCGAAGGCTTGCTCAAGTCCAGGTATGGCTTACGAGTATCCTC    240
SCNU-C-3470
GAGTTTGCTGGACATCGTCGAAGGCTTGCTCAAGTCCAGGTATGGCTTACGAGTATCCTC    240
SCNU-C-3328
GAGTTTACTGGACATCGTCGAAGGCTTGCTCAAGTCCAGGTATGGCTTACGAGTATCCTC    240
******.*****************************************************

Bol031422
ACAATGGAAAACCAATATAATGAGCTTCTTAATACTAGTGAGGTTGAGCTTCAAAGGTTG    300
SCNU-C-3470
ACAATGGAAAACCAATATAATGAGCTTCTTAATACTAGTGAGGTTGAGCTTCAAAGGTTG    300
```

SCNU-C-3328
ACAATGGAAAACCAATATAATGAGCTTCTTAATACTAGAGACGTTGAGCTTCAAAGGTTG 300
*************************************:** ******************

Bol031422
TGTCTCTGTCGATTTTGCTCTAAAAATGTGAAAAAGAGCTATCTTTATGGGAAAAGGGTT 360
SCNU-C-3470
TGTCTCTGTCGATTTTGCTCTAAAAATGTGAAAAAGAGCTATCTTTATGGGAAAAGGGTT 360
SCNU-C-3328
TGTCTCTGTCGATTTTGCTCTAAAAATGTGAAAAAGAGCTATCTTTATGGGAAAAGGGTT 360
****

Bol031422 ATGGTAATGTTGAGGGAGGTTGAGAGTCTTAGTT---CTCAGGGAGAATTTAATGGGGTG
417
SCNU-C-3470 ATGGTAATGTTGAGGGAGGTTGAGAGTCTTAGTT---
CTCAGGGAGAATTTAATGGGGTG 417
SCNU-C-3328
ATGGTAATGTTGAGGGAGGTTGAGAGTCTTATCCGTCAACAACGAGAATTTGATGAGGTG 420
*******************************   .:**. ********.***.****

Bol031422 ACTGATGAAACTCCTATAGCTGAGGGTGAAGAGTTGCCTATTCAACCAACCATAG---GT
474
SCNU-C-3470
ACTGATGAAACTCCTATAGCTGAGGGTGAAGAGTTGCCTATCCAACACACAATTGTTGGT 477
SCNU-C-3328 ACTGACGCAACTCCTATAGCTGAGGGTGAAGAGTTGCCTATTCAACCAACCATAG---
GT 477
***** *.********************************* ****..**.**:*   **

Bol031422
CAGGAAACAATGCTGGAAATGGTATGGAACCGTCTGATGGAAGATGAAGTTGGGATTGTG 534
SCNU-C-3470
CAGGAAACAATGCTGGAAATGGTATGGAACCGTCTGATGGAAGATGAAGTTGGGATTGTG 537
SCNU-C-3328
CAGGAAACAATGCTGGAAATGGTATGGAACCGTCTGATGGAAGATGAAGTTGGGATTGTG 537
****

Bol031422
GGTCTGTACGGTATGGGGGGAGTTGGCAAAACCACCCTTCTCACGCAGATCAACAATAGG 594
SCNU-C-3470
GGTCTGTACGGTATGGGGGGAGTTGGCAAAACCACCCTTCTCACGCAGATCAACAATAGG 597
SCNU-C-3328
GGTCTGTACGGTATGGGTGGAGTTGGCAAAACTACCCTTCTCACGCAGATCAACAATAGG 597
***************** ************** ***************************

Bol031422
TTTTCTGATAGAGGGCATGGGTTCGGTGTTGTGATATGGGTTGTGGTGTCTCAAAATGCA 654
SCNU-C-3470
TTTTCTGATAGAGGGCATGGGTTCGGTGTTGTGATATGGGTTGTGGTGTCTCAAAATGCA 657
SCNU-C-3328
TTTTCTAAAAGAGGTGGTGGGTTTGATGTTGTGATATGGGTTGTGGTGTCACAAAATGCA 657
******.*.*****   .****** *.************************.*********

Bol031422
ACAGTCCATAAGATTCAAGGGAGCATTGGTGAAAAGCTAGGCCTTGGGGGGAAAGAGTGG 714
SCNU-C-3470
ACAGTCCATAAGATTCAAGGGAGCATTGGTGAAAAGCTAGGCCTTGGGGGGAAAGAGTGG 717

SCNU-C-3328
ACAGTCCATAAGATTCAAGGGAGCATCGGTGAAAAGCTAGGGCTTCTGGGGAAAGAGTGG 717
************************** ************** ***   *************

Bol031422
GAGGAGAAAAGAGAGATGAAGAGAGGCCAAGACATCCACAACGTTCTTAGGAAGAAGAAG 774
SCNU-C-3470
GAGGAGAAAAGTGAGATGAAGAGAGGCCAAGACATCCACAACGTTCTTAGGAAGAAGAAG 777
SCNU-C-3328
GACGAGAAAAGTGAGATGAAGAGAGGCCAAGACATCCACAACGTTCTTAGGAAGAAGAAG 777
** ********:**

Bol031422
GTTGTGTTATTGTTGGATGATATATGGGAGAAAGTGAATTTAAGTACGATTGGAGTCCCA 834
SCNU-C-3470
TTTGTGTTATTGTTGGATGATATATGGGAGAAAGTGAATTTAAGTACGATTGGAGTCCCA 837
SCNU-C-3328
TTTGTGTTATTGTTGGATGATATATGGGAGAAAGTGAATTTAAGTACGATTGGAGTCCCA 837
 ****

Bol031422
TATCCGAGCAAGGTAAATGGAAGCAAAGTAGTATTTACCACACGCTCTCGAGATGTGTGT 894
SCNU-C-3470
TATCCGAGCAAGGTAAATGGAAGCAAAGTAGTATTTACCACACGCTCTCGAGATGTGTGT 897
SCNU-C-3328
TATCCGAGCAAAGTAAATGGAAGCAAAGTAGCATTTACCACACGCTCTCGAGATGTGTGT 897
***********.******************* ****************************

Bol031422
GGGCGCATGGGGGTTGATGACCCGATCGAAGTCCGTTGTCTGGACACGGACAAAGCCTGG 954
SCNU-C-3470
GGGCGCATGGGGGTTGATGACCCGATCGAAGTCCGTTGTCTGGACACGGACAAAGCCTGG 957
SCNU-C-3328
GGACGCATGGAGGTTGATGATCCGATCGAAGTCCGTTGTCTGGACACGGACAAAGCCTGG 957
.*****.********* ***************************************

Bol031422
GATTTGTTCAAAGAGAAAGTCGGAGAACACACACTGGGAAGGCACCCAGACATTCCCGAG 1014
SCNU-C-3470
GATTTGTTCAAAGAGAAAGTCGGAGAACACACACTGGGAAGGCACCCAGACATTCCCGAG 1017
SCNU-C-3328
GATTTGTTCAAAAAGAAAGTCGGGGAAAACACGTTGGGAAGCCACCCAGGCATTCCCGAG 1017
************.**********.***.****. ******* *******.**********

Bol031422
CACGCAAGGAAAGTCGCAGGGAAATGTCGTGGCCTACCATTGGCACTTAATGTCATCGGC 1074
SCNU-C-3470
CACGCAAGGAAAGTCGCAGGGAAATGTCGTGGCCTACCATTGGCACTTAATGTCATCGGC 1077
SCNU-C-3328
CTCGCAAGGGAAGTCGCTGGGAAATGTCGTGGCCTACCATTGGCCCTTAATGTCATCGGT 1077
*.*******.*******.**************************.**************

Bol031422 GAAACAATG--- 1083
SCNU-C-3470 GAAACAATG--- 1086
SCNU-C-3328
GGAACAATG**GCGAGCAAAAGATCACTACAAGAGTGGCGTCTAGCAGTTGATGTTTTGACT** 1137

* . *******

Bol031422 -- 1083
SCNU-C-3470 -- 1086
SCNU-C-3328
TCATCTGCCAGAGAGTTTTCAGGCGTGGAAGATGAGATTCTTCAGATATTGAAGTATAGC 1197

Bol031422 -- 1083
SCNU-C-3470 -- 1086
SCNU-C-3328
TACGATAGTTTGGATGGTGAGATGACCAAGTCATGTTTTCTGTATTGCTCTCTGTTTCCT 1257

Bol031422 -- 1083
SCNU-C-3470 -- 1086
SCNU-C-3328
GAAGATGACATTATTGATAAAGAAGAACTGATAGAGTATTGGATAGGTGAGGGGTTCATT 1317

Bol031422 -- 1083
SCNU-C-3470 -- 1086
SCNU-C-3328
GATGAGAAGGAAGGAAGAGAAATGGCAATGAACAAAGGTTATGAGATACTCGAGACCCTT 1377

Bol031422 -TCCGTGCATGTTTACTGTTGGAAGATGACGAGGATGAATCAGAAGTGA
1131
SCNU-C-3470 -TCCGTGCATGTTTACTGTTGGAAGATGACGAGGATGAATCAGAAGTGA
1134
SCNU-C-3328 GTCCGTGCATGTTTGTTGTTGGAAGATGACGAGGATGAATCAGAAGTGA
1426
*************. **********************************

(B)

- Protein sequence

>Bol031422 (Protein) Reference
MGGCFSVSVSCDQVVNQVSQCLCLNGSYIHNLPQNLGTLHKAMRALKAKRDDV
QGRVDREEFAGHRRRLAQVQVWLTSILTMENQYNELLNTSEVELQRLCLCRFCSK
NVKKSYLYGKRVMVMLREVESLSSQGEFNGVTDETPIAEGEELPIQPTIGQETMLE
MVWNRLMEDEVGIVGLYGMGGVGKTTLLTQINNRFSDRGHGFGVVIWVVVSQN
ATVHKIQGSIGEKLGLGGKEWEEKREMKRGQDIHNVLRKKKVVLLLDDIWEKVN
LSTIGVPYPSKVNGSKVVFTTRSRDVCGRMGVDDPIEVRCLDTDKAWDLFKEKVG
EHTLGRHPDIPEHARKVAGKCRGLPLALNVIGETMSVHVYCWKMTRMNQK

>SCNU-C-3470 (Protein) Resistant line
MGGCFSVSVSCDQVVNQVSQCLCLNGSYIHNLPQNLGTLHKAMRALKAKRDDV
QGRVDREEFAGHRRRLAQVQVWLTSILTMENQYNELLNTSEVELQRLCLCRFCSK
NVKKSYLYGKRVMVMLREVESLSSQGEFNGVTDETPIAEGEELPIQHTIVGQETML
EMVWNRLMEDEVGIVGLYGMGGVGKTTLLTQINNRFSDRGHGFGVVIWVVVSQ
NATVHKIQGSIGEKLGLGGKEWEEKSEMKRGQDIHNVLRKKKFVLLLDDIWEKV
NLSTIGVPYPSKVNGSKVVFTTRSRDVCGRMGVDDPIEVRCLDTDKAWDLFKEKV
GEHTLGRHPDIPEHARKVAGKCRGLPLALNVIGETMSVHVYCWKMTRMNQK

>SCNU-C-3328 (Protein) Susceptible line
MGGCFSVSLSCDQVVNQVFRCLCLKGSYVHNLPQNLASLEKAMGTLTAKRDDV
QGRVDREEFTGHRRRLAQVQVWLTSILTMENQYNELLNTRDVELQRLCLCRFCS
KNVKKSYLYGKRVMVMLREVESLIRQQREFDEVTDATPIAEGEELPIQPTIGQETM
LEMVWNRLMEDEVGIVGLYGMGGVGKTTLLTQINNRFSKRGGGFDVVIWVVVS
QNATVHKIQGSIGEKLGLLGKEWDEKSEMKRGQDIHNVLRKKKFVLLLDDIWEK
VNLSTIGVPYPSKVNGSKVAFTTRSRDVCGRMEVDDPIEVRCLDTDKAWDLFKKK
VGENTLGSHPGIPELAREVAGKCRGLPLALNVIGGTMASKRSLQEWRLAVDVLTS
SAREFSGVEDEILQILKYSYDSLDGEMTKSCFLYCSLFPEDDIIDKEELIEYWIGEGFI
DEKEGREMAMNKGYEILETLVRACLLLEDDEDESEV

- Protein alignment

Bol031422 MGGCFSVSVSCDQVVNQVSQCLCLNGSYIHNLPQNLGTLHKAMRALKAKRDDVQGRVDRE
60

SCNU-C-3470 MGGCFSVSVSCDQVVNQVSQCLCLNGSYIHNLPQNLGTLHKAMRALKAKRDDVQGRVDRE 60
SCNU-C-3328 MGGCFSVSLSCDQVVNQVFRCLCLKGSYVHNLPQNLASLEKAMGTLTAKRDDVQGRVDRE 60
********:********* :****:***:*******.:*.*** :*.*************

Bol031422 EFAGHRRRLAQVQVWLTSILTMENQYNELLNTSEVELQRLCLCRFCSKNVKKSYLYGKRV 120
SCNU-C-3470 EFAGHRRRLAQVQVWLTSILTMENQYNELLNTSEVELQRLCLCRFCSKNVKKSYLYGKRV 120
SCNU-C-3328 EFTGHRRRLAQVQVWLTSILTMENQYNELLNTRDVELQRLCLCRFCSKNVKKSYLYGKRV 120
:*************************** :**************************

Bol031422 MVMLREVESLS-SQGEFNGVTDETPIAEGEELPIQPTIG-QETMLEMVWNRLMEDEVGIV 178
SCNU-C-3470 MVMLREVESLS-SQGEFNGVTDETPIAEGEELPIQHTIVGQETMLEMVWNRLMEDEVGIV 179
SCNU-C-3328 MVMLREVESLIRQQREFDEVTDATPIAEGEELPIQPTIG-QETMLEMVWNRLMEDEVGIV 179
**********   .* **: *** ************ **   ********************

Bol031422 GLYGMGGVGKTTLLTQINNRFSDRGHGFGVVIWVVVSQNATVHKIQGSIGEKLGLGGKEW 238
SCNU-C-3470 GLYGMGGVGKTTLLTQINNRFSDRGHGFGVVIWVVVSQNATVHKIQGSIGEKLGLGGKEW 239
SCNU-C-3328 GLYGMGGVGKTTLLTQINNRFSKRGGGFDVVIWVVVSQNATVHKIQGSIGEKLGLLGKEW 239
**********************.** **.************************** ****

Bol031422 EEKREMKRGQDIHNVLRKKKVVLLLDDIWEKVNLSTIGVPYPSKVNGSKVVFTTRSRDVC 298
SCNU-C-3470 EEKSEMKRGQDIHNVLRKKKFVLLLDDIWEKVNLSTIGVPYPSKVNGSKVVFTTRSRDVC 299
SCNU-C-3328 DEKSEMKRGQDIHNVLRKKKFVLLLDDIWEKVNLSTIGVPYPSKVNGSKVAFTTRSRDVC 299
:** ****************.*****************************.*********

Bol031422 GRMGVDDPIEVRCLDTDKAWDLFKEKVGEHTLGRHPDIPEHARKVAGKCRGLPLALNVIG 358
SCNU-C-3470 GRMGVDDPIEVRCLDTDKAWDLFKEKVGEHTLGRHPDIPEHARKVAGKCRGLPLALNVIG 359
SCNU-C-3328 GRMEVDDPIEVRCLDTDKAWDLFKKKVGENTLGSHPGIPELAREVAGKCRGLPLALNVIG 359
*** ********************:****.*** **.*** **:****************

Bol031422 ETMSVHVYCWKMTRMNQK-- 376
SCNU-C-3470 ETMSVHVYCWKMTRMNQK-- 377
SCNU-C-3328 GTMASKRSLQEWRLAVDV**LTSSAREFSGVEDEILQILKYSYDSLDGEMTKSCFLYCSLFP** 419
**: : : :

Bol031422 -- 376
SCNU-C-3470 -- 377
SCNU-C-3328 **EDDIIDKEELIEYWIGEGFIDEKEGREMAMNKGYEILETLVRACLLLEDDEDESEV** 475

Figure S3. Sequence information obtained by cloning and sequencing of cabbage resistant (SCNU-C-3470) and susceptible (SCNU-C-3328) lines for a NBS-LRR gene (*Bol031422*) and their alignments. Genomic DNA (gDNA) sequence and their alignment (A), protein sequence and their alignment (B). Sequence alignments along with the references sequences retrieved from Bolbase (http://www.ocri-genomics.org/bolbase/) considering *Brassica oleracea* as reference genome database. In sequence alignment asterisks (*) indicate sequence similarity. Absence of asterisks indicates sequence dissimilarity. Endesh (-) indicates insertion/deletion of nucleotides. The grey and red highlights in the alignment of genomic DNA sequences indicate the BR6-InDel-F and BR6-InDel-R, respectively. The red text colors in the alignment of genomic sequences indicate InDel regions.
