# Supplementary material for: Inheritance of Black Rot Resistance and Development of Molecular Marker Linked to Xcc Races 6 and 7 Resistance in Cabbage
Source: Plants (Basel). 2021 Sep 17;10(9):1940. doi: 10.3390/plants10091940 (PMC8472523; doi:10.3390/plants10091940)
Supplement: Supplementary file 1 [file plants-10-01940-s001.zip › Table S1.pdf]

Table S1. Primer specifications of candidate Black rot resistance NBS gene *Bol031422*.

Gene ID	Forward sequence (Primer-F)	Reverse sequence (Primer-R)	TM (℃)	Product Size
Bol031422	ATGGGAGGTTGTTTCTCTGTCTCA	AGATGACGAGGATGAATCAGAAGTGA	58	1134 bp
