# Supplementary material for: Inheritance of Black Rot Resistance and Development of Molecular Marker Linked to Xcc Races 6 and 7 Resistance in Cabbage
Source: Plants (Basel). 2021 Sep 17;10(9):1940. doi: 10.3390/plants10091940 (PMC8472523; doi:10.3390/plants10091940)
Supplement: Supplementary file 1 [file plants-10-01940-s001.zip › Table S3.pdf]

Table S3. Comparison of bioassay and molecular screening results for black rot resistance in cabbage inbred lines. S; susceptible, H; hetero, R; resistant

Sl. No.	Lines	Race 6	BR6-InDel	Race 7	BR6-InDel
		Phenotype	Genotype	Phenotype	Genotype
1	SCNU-C-106	S	H	S	H
2	SCNU-C-107	S	H	R	H
3	SCNU-C-3122	S	R	S	R
4	SCNU-C-3270	S	H	R	H
5	SCNU-C-3273	S	S	-	S
6	SCNU-C-3305	S	H	S	H
7	SCNU-C-3328	S	S	S	S
8	SCNU-C-3412	S	S	S	S
9	SCNU-C-3414	S	H	-	H
10	SCNU-C-3449	S	S	S	S
11	SCNU-C-3470	R	R	R	R
12	SCNU-C-3631	S	H	S	H
13	SCNU-C-3637	S	H	S	H
14	SCNU-C-3639	S	S	S	S
15	SCNU-C-4059	S	S	R	S
16	SCNU-C-4066	S	H	S	H
17	SCNU-C-4071	S	S	S	S
18	SCNU-C-4072	S	S	S	S
19	SCNU-C-4073	S	H	S	H
20	SCNU-C-4074	S	H	S	H
21	SCNU-C-4085	S	H	S	H
22	SCNU-C-4105	S	R	S	R
23	SCNU-C-4118	R	H	S	H
24	SCNU-C-4161	S	H	S	H
25	SCNU-C-4168	S	H	S	H
26	SCNU-C-4302	S	S	S	S
27	SCNU-C-4320	S	H	S	H
Matched			23.0		19.0
Not matched			4.0		6.0
% Adaptability			85.2		76.0
