# Supplementary figures and images for: Inheritance of Black Rot Resistance and Development of Molecular Marker Linked to Xcc Races 6 and 7 Resistance in Cabbage
Source: Plants (Basel). 2021 Sep 17;10(9):1940. doi: 10.3390/plants10091940 (PMC8472523; doi:10.3390/plants10091940)

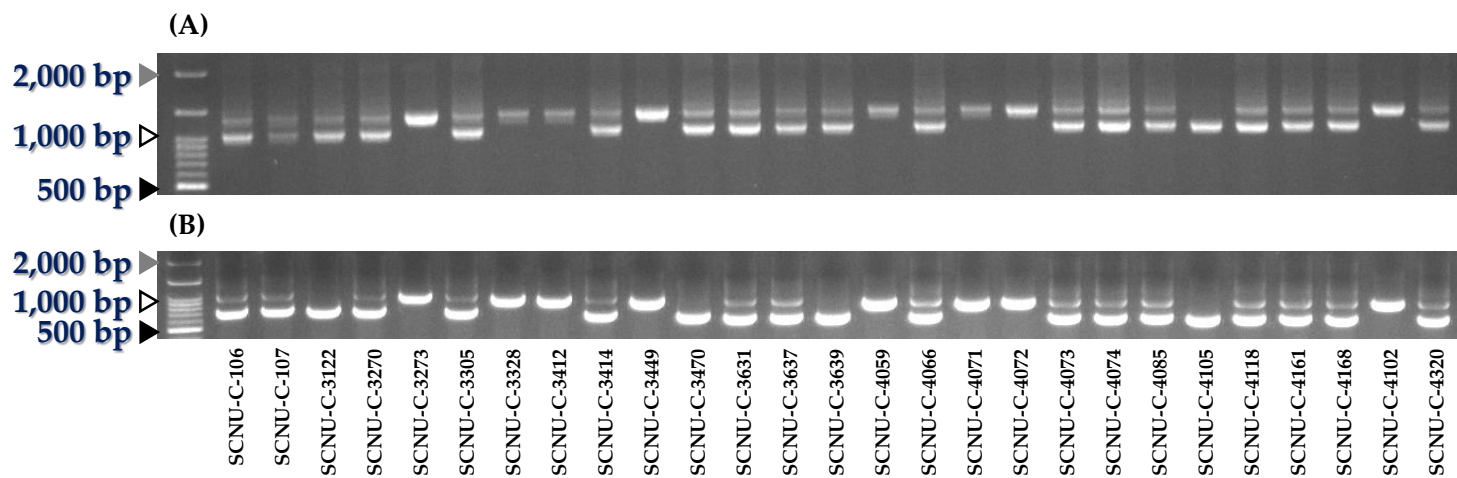

**Figure S4.** Banding profile of *R* gene (Bol031422) in 27 inbred cabbage lines using Pimer-F/R (A), BR6-InDel-F/R (B).

Supplement: Supplementary file 1 [file plants-10-01940-s001.zip › Figure S4.pdf]
